# Greenspace exposure and associated health outcomes: an updated systematic review of reviews

**DOI:** 10.12688/f1000research.166852.3

**Published:** 2026-11-05

**Authors:** Brittnee Bryer, Nicholas J Osborne, Jialu Wang, Rajarshi Dasgupta, Gail Williams, Darsy Darssan

**Affiliations:** 1 The University of Queensland School of Public Health, Herston, Queensland, Australia; 2 School of Population Health, University of New South Wales, Sydney, New South Wales, Australia; 3 European Centre for Environment and Human Health, University of Exeter Medical School, Exeter, England, UK; 4 Queensland University of Technology School of Public Health and Social Work, Kelvin Grove, Queensland, Australia; 5 School of Public Policy, Indian Institute of Technology, Delhi, New Delhi, India

**Keywords:** Human health, natural environment, outdoor environment, umbrella review, biodiversity, greenery, urbanisation

## Abstract

**Background:**

Studies, including umbrella reviews, have provided evidence linking greenspace exposure to specific health outcomes. To address methodological limitations in existing umbrella reviews, this umbrella review aims to expand knowledge on the association between greenspace and human health by summarising evidence from both quantitative and qualitative systematic reviews and meta-analyses.

**Methods:**

This umbrella review builds upon our previous review with updates to reflect recent developments in the field. Five databases (PubMed, Embase, the Cumulative Index to Nursing and Allied Health Literature [CINAHL], Scopus, and the Cochrane Database of Systematic Reviews) were searched for articles published in English in peer-reviewed journals between December 2020 and June 2024. Systematic reviews and meta-analyses were included if they clearly defined measures of greenspace exposure and reported health outcomes directly attributable to greenspace exposure (PROSPERO: CRD42022383421). The methodological quality and risk of bias of each included review were evaluated by two independent reviewers.

**Results:**

A total of 45 articles were included in this umbrella review. Greenspace exposure was beneficially associated with mental health, general health and quality of life, and cardiovascular and metabolic health. Inconsistent or inconclusive associations were observed for respiratory health and allergies, maternal health and birth outcomes, cancer, and cognitive function. Some harmful associations were reported for communicable and vector-borne diseases, although these were based on a limited number of systematic reviews. Most of the included reviews had a high risk of bias and critically low methodological quality.

**Discussion:**

This umbrella review highlights the multifaceted health benefits of greenspace exposure while underlining existing evidence gaps and methodological challenges. The results indicate that increasing access to greenspace could be a simple and cost-effective strategy to improve population health and reduce health inequalities.

## Introduction

The rapid pace of urbanisation and environmental change has escalated interest in understanding how natural environments impact human health ( Farkas et al., 2023; Wang et al., 2024). Urban greenspaces, encompassing parks, forests, and other vegetated areas, have emerged as crucial components of urban ecosystems, offering potential health benefits ( Yang et al., 2021). Studies, including umbrella reviews, have provided evidence linking greenspace exposure to specific health outcomes, such as mental ( Bonaccorsi et al., 2023; Cuijpers et al., 2023), cognitive ( Zare Sakhvidi et al., 2023), and cardiometabolic health ( Liu et al., 2023; Sharifi et al., 2024), as well as birth outcomes ( Khalaf et al., 2025; Zare Sakhvidi et al., 2023), mortality ( Bryer et al., 2024; Song et al., 2024), and quality of life (QoL) ( Bonaccorsi et al., 2023). However, limitations in the methodology of these reviews have resulted in some gaps in the literature.

Many systematic reviews struggle to quantitatively synthesise evidence on the association between greenspace and individual health outcomes due to the limited number of original studies available. In such cases, these reviews often present their findings narratively. Conversely, in 2024, Xie et al. (2024) conducted an umbrella review examining the credibility of evidence on the association between greenspace and human health. While the authors graded the credibility of the current evidence, they focused solely on quantitative results. When conducting a systematic review on the association between greenspace and human health, authors should consider both quantitative and qualitative results. This will provide a more comprehensive understanding of the association and enhance the generalisability of the findings.

Recent years have also seen the emergence of new umbrella reviews on the greenspace-health association. However, these reviews have typically focused on a narrower selection of health domains or demographic groups. For instance, some reviews explored the association between greenspace and specific health outcomes, such as cardiovascular disease (CVD) ( Liu et al., 2023) and mental health ( Cuijpers et al., 2023). Others have concentrated on specific age groups, including children and adolescents ( Zare Sakhvidi et al., 2023) and older adults ( Bonaccorsi et al., 2023).

There is a clear need for an updated and comprehensive review that outlines both quantitative and qualitative evidence while encompassing all health outcomes across all age groups. To address this gap, this umbrella review aims to expand existing knowledge on the association between greenspace and human health by summarising evidence from both quantitative and qualitative systematic reviews and meta-analyses.

## Methods

This umbrella review builds upon our previous review (January 2010 – December 2020) ( Bryer et al., 2024) with updates to reflect recent developments in the field. The protocol was registered with the International Prospective Register of Systematic Reviews (PROSPERO; registration ID: CRD42022383421) ( Bryer et al., 2022). The review adheres to the Preferred Reporting Items for Systematic Reviews and Meta-Analyses (PRISMA) guidelines ( Page et al., 2021).

### Search strategy

The existing search strategy from our previous review was used to identify peer-reviewed systematic reviews and meta-analyses examining the association between greenspace exposure and human health. The search was restricted to studies conducted in humans published between December 2020 and June 2024, including reviews first published online during this period (the full search strategy for each database is available in the extended data: Supplementary Table S1). Searches were conducted on July 1, 2024 across five databases: PubMed, Embase, the Cumulative Index to Nursing and Allied Health Literature (CINAHL), Scopus, and the Cochrane Database of Systematic Reviews. EndNote was used for reference management ( The EndNote Team, 2013).

### Eligibility criteria

Two authors independently screened the titles and abstracts and full texts of identified systematic reviews. Inclusion criteria were as follows: i) systematic review or meta-analysis; ii) published in English in a peer-reviewed journal; iii) greenspace exposure clearly defined using objective or subjective measures; iv) reported health outcome(s) directly attributable to greenspace exposure (e.g., independent of blue space). Reviews were excluded if they were: i) systematic reviews that did not adhere to standardised systematic review methodologies (e.g. lacking database search details, search terms, or quality assessment); ii) scoping reviews; iii) reviews that combined greenspace effects with blue space; iv) studies that only reported on determinants of health (e.g. body mass index [BMI], physical activity, social health). Disagreements during screening were resolved through discussions with the last author. Covidence software was used to conduct all stages of screening ( Covidence systematic review software, 2022).

### Data extraction

Two authors independently extracted data using a pre-established data extraction form ( Bryer et al., 2024). Extracted data included: design of original studies, types and measures of greenspace exposures, health outcomes, and the main findings of the review. Any discrepancies in extracted data were resolved through discussions with the last author.

### Analysis criteria

The objectives, greenspace measures, health outcomes, and findings of each included review were summarised qualitatively. Consistent with the purpose of umbrella reviews, which outline evidence from systematic reviews rather than re-synthesising the results, no meta-analysis was performed ( Aromataris et al., 2015; Pollock et al., 2024). Where quantitative data were available, effect estimates and 95% confidence intervals were reported.

The total number of original studies, classified by study design, was recorded, along with details of greenspace measures and reported health outcomes. For each health outcome, the corresponding International Classification of Diseases, 10 ^th^ Edition (ICD-10) code(s) was also noted ( World Health Organization, 2019). The corrected coverage area (CCA) was calculated for each health outcome and outcome category. CCA values were classified according to Pieper et al. (2014).

Methodological quality and risk of bias were independently evaluated by two authors using A MeaSurement Tool to Assess systematic Reviews 2 (AMSTAR-2) ( Shea et al., 2017) and the Risk of Bias Assessment Tool for Systematic Reviews (ROBIS) ( Whiting et al., 2016), respectively. Disagreements were resolved through discussions with the last author. Systematic reviews were not excluded based on methodological quality or risk of bias.

## Results

### Study selection

The search strategy identified 1,981 unique articles, of which 277 were retained for full-text screening. Ultimately, 45 articles met the inclusion criteria for this umbrella review. The PRISMA flowchart is available under the Reporting guidelines section at the end of this article. 

### Study type and characteristics

Most included systematic reviews were published in 2022 (n = 18, 40.0%). The number of original studies within these reviews ranged from two to 140, with participant sample sizes spanning from two to 159 million ( Table 1). Various study designs were represented, with cross-sectional studies being the most prevalent (n = 501, 45.7%) ( Table 2).

**Table 1.** Characteristics of included systematic reviews *.

Author (year)	No. original studies (location)	Type of study (n)	Objective(s) of the study	Sample size (age range)	Greenspace measures	Outcome measures	Main finding(s)
Aarthi et al. (2023)	3 (Asia)	Cross-sectional (3)	Review associations of built environment characteristics with T2DM in Asia.	4,155-341,211 (0-99)	NDVI, SAVI, greenspace ratio, green vision index, evergreen tree configuration	T2DM, glucose levels	Greenness associated with lower glucose levels in Taiwan and China.
Ahmer et al. (2024)	31 (North America, Europe, Asia, Oceania)	Cross-sectional (26), cohort (3), case-control (1), quasi-experimental (1)	Review influence of residential greenspaces on birth outcomes of pregnant women.	301-3,753,788 (unclear)	NDVI, greenspace/land percentage, tree canopy coverage, LiDAR	BW, LBW, PTB, SGA	Residential greenspaces positively associated with increased BW and lower odds of LBW, PTB, and SGA deliveries.
Batterham et al. (2022)	4 (North America, Europe, Asia, Oceania)	Cross-sectional (4)	Synthesise quantitative evidence for relationship between environmental factors and mental health in rural areas.	2,020-33,823 (unclear-89)	Percentage of greenspace type, land cover classes, proportion of forest & agricultural areas, total greenspace ratio	General mental health, anxiety, depression, affective disorders, schizophrenia	Areas with greater greenspace might have better well-being, marginally less depression, and reduced hospitalisations.
Bianconi et al. (2023)	36 (North America, South America, Europe, Asia, Oceania)	Ecological (15), cohort (13), cross-sectional (7), case-control (1)	Assess impacts of urban greenery on cardiovascular and cerebrovascular disease morbidity and mortality.	469-43,000,000 (unclear-89)	NDVI, SAVI, LAI, greenspace percentage/urban park percentage, distance from city parks larger than 1 hectare, tree canopy percentage	CVD mortality, IHD mortality, stroke mortality, CHD, stroke, CVD, AMI, HF, heart disease, IHD	Most studies showed greater greenspace exposure was correlated with better cardiovascular and cerebrovascular health in urban settings.
Bolanis et al. (2024)	23 (North America, Europe, Asia, Africa, Oceania)	Ecological (14), cross-sectional (4), longitudinal (3), pre-post design (1), randomised cross-over (1)	Review evidence on association between greenspace exposure and suicide-related outcomes.	15-8,741,021 (11-77)	NDVI, land cover map, greenspace percentage, number of parks per 1000 people, per capita park area, greenspace fragmentation, mean green patch area, green patch distance, percentage tree cover, tree canopy coverage, activities in forest settings, mountain hiking, decrease time in greenspace	Suicide mortality, self-harm, suicidal ideation	Greenspace exposure may be beneficially associated with suicide-related outcomes, particularly among females.
Briggs et al. (2022)	20 (North America, Europe, Asia)	RCT (20)	Evaluate effectiveness of group-based gardening interventions for increasing well-being and reducing mental ill-health in adults.	20-89 (18-78)	Gardening interventions	Depression, anxiety, stress, QoL, overall mental health, mood, affect, PTSD, burnout	Mixed evidence for the effectiveness of group-based gardening interventions for mental health and wellbeing.
Browning et al. (2022)	37 (North America, Europe)	Cross-sectional (29), longitudinal (8)	Consolidate evidence on how urbanicity modifies relationship between greenspace and health.	1,542-97,574,613 (0-85)	NDVI, green landcover, public greenspace, distance to greenspace, distance-based geographic area, container-based geographic area	Respiratory health, cardiovascular health, diabetes, mortality, cancer, birth outcomes	More analyses showed stronger protective associations in urban areas than in less urban areas.
Cao et al. (2023)	14 (North America, Europe, Asia, Africa, Oceania)	Cohort (10), cross-sectional (3), ecological (1)	Determine association between greenness and AR in children and adolescents.	522-642,313 (0-17)	NDVI, EVI, greenspace coverage, MODIS, green score (residential greenness and neighbourhood green land use)	AR	Results indicate no association between greenness exposure and AR in children and adolescents.
Ccami-Bernal et al. (2023)	13 (North America, Europe, Asia, Oceania)	Cohort (13)	Summarise evidence on exposure to greenspace and incidence of T2DM from longitudinal studies.	1,700-1,922,545 (15-87)	NDVI, forest percentage, percentage of any greenspace, percentage of neighbourhood greenness, EVI, proportion of greenspace within1000m of the residence, VCF	T2DM	Higher exposure to greenspace associated with decreased incidence of T2DM.
Chae et al. (2021)	13 (Asia)	Pre-post (10), RCT (3)	Analyse effects of forest therapy on immune function.	11-61 (unclear-80)	Forest therapy	Immune function	Forest therapy programs may contribute to the improvement of immune function.
Chen et al. (2022)	2 (North America, Europe, Asia, Oceania)	Qualitative (1), mixed methods (1)	Identify types, benefits, motivations, and drawbacks of environmental volunteerism.	5-19 (unclear-92)	Greening volunteerism	Depression, positive affect, distress	Environmental volunteerism can be considered a good model for productive aging and nature conservation.
De la Fuente et al. (2021)	19 (North America, Europe, Asia, Oceania)	Cross-sectional (12), cohort (6), longitudinal (1)	Know the magnitude of effect of greenspace on prevalence of T2DM through analysis of recent evidence.	76-3,920,000 (15-85)	Greenspace exposure by postcode, NDVI, area of parks in neighbourhood, density of trees & park area, frequency & intensity of exposure to nature, street tree density, greenspace satisfaction, distance to nearest park	T2DM	Greater greenspace exposure associated with lower risk of T2DM in the urban context.
Gao et al. (2024)	16 (North America, Europe, Asia)	Cross-sectional (6), cohort (5), ecological (2), case-control (1), RCT (1) non-RCT (1)	Investigate impact of greenspace on COPD-related disease outcomes and summarise reasons for heterogeneity.	18-2,185,170 (unclear)	NDVI, green land cover, tree canopy cover, forest cover, MSAVI, forest bathing	COPD	Greenery may positively impact COPD-related health outcomes, although some studies have yielded different results.
Hsueh et al. (2022)	3 (North America, Europe, Oceania)	Mixed methods (2), qualitative (1)	Synthesise literature on community place-based interventions addressing loneliness and mental health problems.	13-56 (9-98)	Community gardening, exposure to greenery	Depression, anxiety, PTSD, overall mental health	Insufficient evidence of an association between community-based interventions and loneliness or mental health problems.
Kang et al. (2022)	6 (Europe, Asia)	RCT (6)	Overview of RCTs on effects of forest-based interventions on mental health.	33-84 (unclear-79)	Forest therapy, forest bathing, forest videos, forest walking	Depression, anxiety, positive emotions, negative emotions, overall mental health	Forest-based interventions improved mental health of participants in the intervention groups compared to those in the control groups.
Lampert et al. (2021)	8 (North America, Europe, Asia)	Cross-sectional (8)	Synthesise the literature about physical and mental health outcomes associated with community gardening.	50-332 (25-88)	Community gardening	Overall health, number of chronic conditions, stress, anxiety, depression, QoL, PTSD, overall mental wellbeing	Community gardens are associated with health gains for their users, irrespective of age, being an affordable and efficient way of promoting physical and mental health and well-being.
Li et al. (2023)	14 (North America, Europe, Asia)	Cohort (14)	Integrate evidence from cohort studies to obtain best available epidemiological evidence on greenspace exposure and cancer.	7,300-10,481,566 (unclear)	NDVI, proximity to greenspace	All-site cancer, prostate cancer, skin cancer, colorectal cancer, breast cancer, lung cancer, bladder cancer, mouth cancer, other cancer, all-site cancer mortality, lung cancer mortality, prostate cancer mortality	Greenspace exposure reduces lung cancer and prostate cancer mortality, and there may be a potentially beneficial association for prostate, lung, and breast cancer incidence.
Li and Lange (2023)	15 (North America, Europe, Asia, Oceania)	Cross-over (6), RCT (3), cross-sectional (2), pre-post design (2), real-time (1), cohort (1)	Provide a comprehensive analysis of impact of urban landscapes and interventions on stress reduction.	11-164 (14-85)	Percentage land use, percentage of trees, residential gardens, natural/green corridor, semi-natural/natural areas, private greenspace, recreation civic space, parks, window green view, street greenery, landscape elements	Psychological stress, mood, mental wellbeing, cognition, physiological stress, overall health	Suggests that urban landscapes can provide therapeutically relevant changes in psychological states, physiological activity, and cognitive functioning.
Masdor et al. (2023)	5 (North America, Europe)	Cohort (5)	Investigate association between exposure to greenspace in adult population and outcomes of colorectal cancer.	19,408-2,441,566 (30-69)	NDVI, garden percentage, natural environment percentage, ecosystem count, recreational facilities & parks count, residential proximity to greenspace, surrounding greenness	Colorectal cancer, colorectal cancer mortality	There is currently no link between colorectal cancer and greenspace.
Patwary et al. (2024)	18 (North America, Europe, Asia, Oceania)	Cross-sectional (8), cohort (7), ecological (3)	Assess association of greenspaces and built environments with MetS risk.	75-49,893 (10-112)	NDVI, EVI, SAVI, VCF, distance to greenspace, tree canopy density, parkland percentage, percentage land use for park & recreation	MetS	Most studies showed beneficial associations between higher greenspace exposure and lower risk of MetS.
Piva et al. (2024)	10 (Asia)	Exploratory (6), RCT (2), non-RCT (2)	Explore physiological and psychological benefits of forest therapies on healthy and elderly populations.	18-88 (60-80)	Forest bathing, forest exercise	HR, HRV, BP, lung function, cortisol level, mood, depression, QoL, stress, cognitive impairment, neurochemical response	Forest therapy interventions are effective in improving physiological, neurochemical, and biological parameters, and psychological well-being.
Qiu et al. (2022)	29 (Europe, Asia)	Crossover RCT (15), parallel RCT (10), non-RCT (4)	Toassess the effects of forest therapy on BP and SCC in urban residents.	11-348 (19-80)	Forest therapy, forest walking, forest viewing	SBP, DBP, SCC	Longer forest therapy programs have greater BP- and SCC-lowering effects than shorter programs.
Rahimi-Ardabili et al. (2021)	73 (Asia)	Cross-sectional (42), cohort (9), RCT (9), ecological (7), case-control (3), before-after study (2), quasi-experimental (1)	Synthesise the emerging evidence on greenspace and health in mainland China.	20-159,000,000 (16-80)	NDVI, SAVI, proximity/frequency of visits to parks, viewing greenery, green walking, percentage green space coverage, time spent in parks, land cover map	Mood, tension-anxiety, depression, fatigue, overall mental health/mental wellbeing, stress, attention, affective states, ADHD, QoL, overall health, diabetes, eczema, number of chronic conditions, BP, hypertension, CHD, cardiovascular health, MetS, CVD, stroke, cognition, asthma, lung cancer, COPD, rhinitis, number of respiratory diseases, all-cause mortality, other causes of mortality, malaria, dysentery, dengue, tuberculosis, BW, miscarriage	Positive associations found between greenspace and mental health, cardiovascular outcomes, and general health.
Ricciardi et al. (2022)	25 (North America, Europe, Asia, Oceania)	Cross-sectional (19), longitudinal (6)	Summarise studies using objective measures of greenspace exposure and cognitive functioning.	108-38,327 (unclear)	NDVI, EVI, VFC, MEDIx, distance to NOE, percentage of parkland, total land area, total soft surface, tree canopy cover, time spent in green spaces, grass/shrub cover, average percent canopy cover	Cognition, memory, attention, executive function	Evidence was suggestive of a beneficial association between greenspace exposure and cognitive functions but was inconsistent across age groups.
Rigolon et al. (2021)	90 (North America, South America, Europe, Asia, Oceania)	Cross-sectional (79), longitudinal (11)	Review whether green space can contribute to limiting health disparities and moving toward health equity.	106-97,574,613 (unclear-111)	Gardening, green land cover, nature-based programs, exposure to public green space	Eczema, asthma, diabetes, prostate cancer, CVD, all-cause mortality, PTB, infant mortality, overall health	Greenspace had greater protective assocations with overall health in low-SES individuals and neighbourhoods than more affluent groups.
Rojas-Rueda et al. (2021)	19 (South America)	Cross-sectional (14), ecological (3), longitudinal (2)	Review epidemiological evidence on green spaces and health outcomes in Latin America.	120-103,000,000 (0-98)	NDVI, frequency/duration of green space exposure, density/proximity to green space, presence of green space, biodiversity index, sustainable development index, park coverage	Depression, overall mental well-being, cognitive functioning, QoL, infant mortality, life expectancy at birth, cardiorespiratory hospital admission, disability, overall health	Most evidence suggests a positive association between green spaces and health in the region.
Siah et al. (2023)	36 (North America, Europe, Asia)	RCT (22), quasi-experimental (7), crossover design (7)	Identify and synthesise evidence on forest bathing and its impacts on individual well-being.	12-585 (18-90)	Forest bathing	Depression, anxiety, QoL, cortisol level, psychosocial well-being, direct-attention, stress, SBP, DBP, HR, HRV, cholesterol, inflammation, blood glucose, CVD risk, pain, disability, working memory, post-stroke fatigue	There is evidence for the beneficial effects of forest bathing on psychological well-being.
Sivak et al. (2021)	22 (North America, South America, Asia)	Cross-sectional (7), quasi-experimental (5), RCT (4), experimental (2), qualitative (3), case-control (1)	Consolidate literature about vacant lots’ effects on human health, summarise findings and identify gaps in existing evidence.	12-49,246 (8-82)	Exposure to vacant lots	Stress, depression, overall mental health, BP, heavy metal contamination, dermatophytosis, toxocara, foodborne pathogens, West Nile Virus, physical injury	Depression, stress, physical activity, relaxation and socialisation in outdoor areas, and HR improved when living near or being within view of a greened vacant lot versus an unmaintained control lot.
Tang et al. (2023)	35 (North America, Europe, Asia)	Cohort (18), cross-sectional (10), ecological (4), case-control (3)	Examine correlation between greenness exposure and chronic respiratory health issues.	478-10,481,566 (0-89)	NDVI	Asthma, AR, COPD, lung cancer, COPD mortality, lung cancer mortality	There is a correlation between higher greenness exposure and a reduced risk of asthma incidence, lung cancer incidence, and COPD mortality.
Tharrey and Darmon (2021)	15 (North America, Europe, Asia)	Cross-sectional (13), post-test only (1), RCT (1)	Review quantitative studies analysing relationship between participation in collective gardens and gardeners’ health status in urban free-living adults in high-income countries.	30-13,133 (18-94)	Community gardening, allotment gardening	Mood, psychological well-being, psychological distress, stress general mental health, BP, lung function, general health	Several studies found a positive association between collective garden participation and physical, mental, or social health, but the results came mostly from cross-sectional studies.
Tu (2022)	19 (North America, Europe, Asia)	RCT (19)	Explore effect of horticultural therapy on mental health.	13-150 (10-95)	Horticultural therapy	Depression, anxiety, stress, PTSD, positive affect, negative affect, overall mental wellbeing, memory	Meta-analysis provided evidence that horticultural therapy has a positive effect on mental health.
Wang et al. (2022)	48 (North America, South America, Europe, Asia, Africa, Oceania)	Cross-sectional (18), cohort (16), ecological (7), case-control (4), longitudinal (3)	Review of evidence to elucidate association between greenness exposure and allergic diseases.	126-10,500,000 (0-73)	NDVI, VCG, EVI, SAVI, GVI, LiDAR, generalised land use database, ArcGIS, land cover classification maps, distance to nearest city part, residential proximity to greenspace, perceptions of greenspace quality, LUG, percentage greenspace, total number of natural land-cover types, plant diversity, tree canopy cover & agricultural cover	Asthma, AR, ARC, AD, food allergies	Exposure to greener environments early in life associated with reduced risk of AR and asthma in childhood.
Wu et al. (2022)	21 (North America, Europe, Asia, Oceania)	Cohort(10), cross-sectional (7), case-control (4)	Review association between greenspace exposure and incidence of asthma and AR.	187-59,754 (0-34)	NDVI, LiDAR, CLC, GVI	Asthma, AR	Insufficient evidence of an association between greenspace exposure and asthma or AR.
Ye et al. (2022)	140 (North America, South America, Europe, Asia, Oceania)	Cross-sectional (83), cohort (40), ecological (8), longitudinal (4), case-control (4), combined cohort cross-sectional (1)	Summarise measures of greenspace, compile evidence of an association between exposure to greenspace and early onset of health outcomes in childhood and adolescence and explore research gaps on how greenspace affects human health at this early stage of life.	61-5,262,265 (0-19)	NDVI, EVI, SAVI, greenspace coverage (rate, proportion, & percentage), proximity to greenspace, perceived greenspace quantity and quality, frequency/duration of greenspace use, street view green index	Depression, anxiety, ADHD, overall mental well-being, autism, stress, psychological distress, QoL, attention, cognitive & motor function, cognitive development, working memory, asthma, rhinitis, AR, lung function, bronchitis, pneumonia, aeroallergen sensitisation, atopic sensitisation, BP, cardiometabolic health, allosteric load, overall health, myopia, astigmatism, intestinal parasites, IBD, leukaemia	Inconsistent results for associations between greenspace and health outcomes in children and adolescents.
Yi et al. (2022)	17 (Europe, Asia)	RCT (17)	Analyse effectiveness of forest therapy for both physiological and psychological areas.	11-144 (unclear-80)	Forest therapy, nature meditation, viewing greenery, green exercise	Depression, anxiety, mood, overall mental health, QoL, BP, HR, physiological stress, overall health	Forest therapy reduced depressive symptoms, but there was insufficient evidence of an association with BP.
Yuan et al. (2021)	22 (North America, Europe, Asia, Oceania)	Cohort (17), cross-sectional (5)	Synthesise evidence from observational studies to assess relationships of greenspace exposure with mortality and cardiovascular outcomes in older individuals.	1,084-5,988,606 (25-unclear)	NDVI, availability of greenspace, number of parks in buffer area, loss of trees, proportion of greenspace, distance to nearest greenspace, greenspaces per inhabitant, frequency/duration of visits to greenspace, tree canopy	CVD, AMI, stroke, heart attack, CHD, IHD, all-cause mortality, non-accidental mortality, cancer mortality, stroke mortality, respiratory disease mortality, circulatory disease mortality, IHD mortality, AMI mortality, CVD mortality, infectious/parasitic disease mortality, CVD mortality, neurodegenerative disease mortality	Some evidence for associations between increased greenness exposure and reduced risk of all-cause and stroke mortality, as well as major CVD outcomes in elderly populations.
Zagnoli et al. (2022)	12 (North America, Europe, Asia, Oceania)	Cross-sectional (4), cohort (4), case-control (1), ecological (1), combined cohort cross-sectional (1), combined cohort case-control (1)	Assess the relationship between environmental greenness and the risk of dementia and cognitive impairment.	2,424-1,737,460 (unclear)	NDVI, land cover, land use, total greenspace, tree canopy	Dementia, Alzheimer's dementia, non-Alzheimer's dementia, ADRD, VaD, PD, MS, cognitive impairment, dementia mortality, neurodegenerative disease mortality	Suggests that environmental greenness may have a non-linear association with dementia.
Zare Sakhvidi, Knobel, et al. (2022a)	29 (North America, Europe, Asia, Oceania)	Cross-sectional (17), longitudinal (11), case-control (1)	Review association between long-term exposure to greenspace and behavioural problems in children and explore sources of heterogeneities.	169-814,689 (2-18)	NDVI, SAVI, MSAVI, duration of greenspace use, percent coverage, distance to greenspace, type of greenspace, quality of greenspace	ADHD, externalising disorders, internalising disorders, hyperactivity, attention Determinants: conduct problems, peer problems, prosocial behaviour, conditional problems	There are beneficial associations between exposure to greenspace with several behavioural outcomes in children.
Zare Sakhvidi, Yang, et al. (2022b)	18 (North America, Europe, Asia, Oceania)	Cross-sectional (8), cohort (6), case-control (4)	Assess evidence on association between exposure to greenspace and cancer incidence, prevalence, and mortality in adults.	1,910-28,600,000 (0-unclear)	NDVI, proximity to greenness, land cover	Skin cancer, colorectal cancer, breast cancer, prostate cancer, lung cancer, non-melanoma cancer, mouth & throat cancer, all cancer, brain tumour, lung cancer mortality, prostate cancer mortality, oesophageal cancer mortality, all cancer mortality	Part of the available literature is suggestive of a possible protective association of greenspace exposure with breast and prostate cancer, while for other cancers the evidence is still very limited and heterogeneous.
Zhang et al. (2021)	26 (North America, South America, Europe, Asia, Oceania)	Cross-sectional (24), longitudinal (2)	Review pathways linking objectively measured greenspace exposure and mental health outcomes.	109-1,930,048 (12-91)	NDVI, greenspace exposure via GIS, national land cover classification database, distance to greenspace, street view greenness, SVG	Depression, anxiety, psychological distress, overall mental wellbeing	Association between greenspace exposure and mental illness mediated by air quality, physical activity, and perceived stress.
Y. Zhang et al. (2024a)	60 (North America, South America, Europe, Asia, Oceania)	Cross-sectional (32), cohort (28)	Explore effects of greenspace exposure on common psychiatric disorders.	322-61,662,472 (2-97)	NDVI, area of greenspace, parks, view of greenery, greenspace accessibility/availability	Depression, anxiety, ADHD, schizophrenia, dementia, psychiatric disorders	Greenspace linked to lower odds of depression, anxiety, dementia, schizophrenia, and ADHD.
Y. D. Zhang et al. (2024b)	20 (North America, South America, Europe, Asia, Oceania)	Cross-sectional (10), non-RCT (5), RCT (2), before-after intervention (2), cohort (1)	Comprehensively synthesise observational and interventional evidence on association between greenspace exposure and human microbiota.	2-9,129 (0-84)	NDVI, EVI, percentage agricultural land & forest, percentage trees & grassland, percentage greenspace, green remediation, natural environment around home, rubbing hand with soil- & plant-based materials, direct exposure to soil & plants, gardening	Gut microbiota, skin microbiota, oral microbiota, nasal microbiota	Most studies showed associations between greater greenspace exposure and increased diversity of gut and skin microbiota.
X. Zhang et al. (2022c)	24 (North America, Europe, Asia)	Randomised crossover (12), non-randomised crossover (7), randomised parallel group (3), 2x2 factorial design (1), single group crossover (1)	Conduct a systematic review of the anxiety-alleviation benefits of exposure to the natural environment	11-498 (unclear-79)	Trail surrounded by trees, roads surrounded by trees, forest exposure, green area at university, park, garden & landscape photographs	Anxiety	Natural environment or pictures and videos of the natural environment can be effective in reducing anxiety.
Zhao et al. (2022)	38 (North America, South America, Europe, Asia, Oceania)	Cross-sectional (27), cohort (9), longitudinal (1), case-control (1)	Review associations between greenspace and BP levels/hypertension.	73-3,920,000 (4-111)	NDVI, SAVI, EVI, greenspace percentage in buffer, tree cover, distance to greenspace, land cover, number of street trees, proportion greenspace, availability of greenspace	Hypertension, SBP, DBP	Higher greenspace exposure was associated with lower SBP and DBP, and lower odds of hypertension.
Zhao et al. (2021)	9 (North America, Europe, Asia)	Cohort (4), case-control (3), cross-sectional (2)	Summarise natural, physical, and social environmental factors associated with cognitive impairment and dementia.	675-1,737,460 (34-101)	NDVI, generalised land use, density of greenspace, percentage of greenspace, distance to greenspace	Dementia, Alzheimer’s dementia, non-Alzheimer’s dementia, cognitive impairment	More residential greenness associated with lower odds of cognitive impairment and dementia.

* AD: atopic dermatitis; ADHD: attention deficit hyperactivity disorder; ADRD: Alzheimer’s disease and related dementia; AMI: acute myocardial infarction; AR: allergic rhinitis; ARC: allergic rhino-conjunctivitis; BMI: body mass index; BP: blood pressure; BW: birthweight; CHD: coronary heart disease; COPD: chronic obstructive pulmonary disease; CLC: coordination of information of the environment (CORINE) land cover; CVD: cardiovascular disease; DBP: diastolic blood pressure; EVI: enhanced vegetation index; GIS: geographic information system; GVI: green view index; HF: heart failure; HR: heart rate; HRV: heart rate variability; IBD: irritable bowel disease; IHD: ischaemic heart disease; LAI: leaf area index; LBW: low birth weight; LiDAR: light detection and ranging; LUG: land use greenness; MEDIx: multiple environmental deprivation index; MetS: metabolic syndrome; MODIS: moderate resolution imaging spectroradiometer; MS: multiple sclerosis; MSAVI: modified soil adjusted vegetation index; NDVI: normalised difference vegetation index; NOE: natural outdoor environment; PD: Parkinson’s disease; PTB: pre-term birth; PTSD: post-traumatic stress disorder; QoL: quality of life; RCT: randomised controlled trial; SAVI: soil-adjusted vegetation index; SBP: systolic blood pressure; SCC: salivary cortisol concentration; SES: socioeconomic status; SGA: small for gestational age; SVG: street-view imagery-based greenness indices; T2DM: type 2 diabetes mellitus; VaD: vascular dementia; VCF: vegetative continuous field; VFC: vegetation fractional cover.

**Table 2.** Study design of original studies in included systematic reviews.

Study design	n (%)
Cross-sectional	501(45.7%)
Cohort	211 (19.2%)
Randomised Controlled Trial (RCT)	109 (9.9%)
Ecological	65 (5.9%)
Longitudinal	52 (4.7%)
Case-control	32 (2.9%)
Other ^a^	127 (11.6%)

^a^ Other: crossover RCT, quasi-experimental, pre-post design, crossover design, randomised crossover, non-RCT, parallel RCT, non-randomised crossover, exploratory, qualitative, before-after study, randomised parallel group, mixed methods, experimental, combined cohort cross-sectional, post-test only, single group crossover, real-time study, randomised controlled crossover, 2x2 factorial design, combined cohort case-control.

### Greenspace exposure

A wide array of greenspace exposure measures were reported ( Table 3). The Normalised Difference Vegetation Index (NDVI), a common quantitative measure of vegetation, appeared in 27 systematic reviews (60.0%). Other frequently used measures included greenspace percentage, proximity to greenspace, presence of tree canopies, Soil Adjusted Vegetation Index (SAVI), Enhanced Vegetation Index (EVI), availability of greenspace, and gardening. Additionally, 37 reviews (82.2%) reported alternative greenspace measures, encompassing both qualitative and quantitative assessments, as well as intervention activities.

**Table 3.** Measures of greenspace exposure in included systematic reviews.

Greenspace exposure measure	Counts of greenspace exposure measure (% out of 45 reviews)	Review articles
NDVI ^b^	27 (60.0%)	Aarthi et al. (2023); Ahmer et al. (2024); Bianconi et al. (2023); Bolanis et al. (2024); Cao et al. (2023); Ccami-Bernal et al. (2023); De la Fuente et al. (2021); Gao et al. (2024); Li et al. (2023); Li and Lange (2023); Masdor et al. (2023); Patwary et al. (2024); Rahimi-Ardabili et al. (2021); Ricciardi et al. (2022); Rojas-Rueda et al. (2021); Tang et al. (2023); Wang et al. (2022); Wu et al. (2022); Ye et al. (2022); Yuan et al. (2021); Zagnoli et al. (2022); Zare Sakhvidi, Knobel, et al. (2022a); Zare Sakhvidi, Yang, et al. (2022b); Zhang et al. (2021); Y. D. Zhang et al. (2024b); Zhao et al. (2022); Zhao et al. (2021)
Greenspace percentage	16 (35.6%)	Ahmer et al. (2024); Batterham et al. (2022); Bianconi et al. (2023); Bolanis et al. (2024); Ccami-Bernal et al. (2023); Li and Lange (2023); Masdor et al. (2023); Patwary et al. (2024); Rahimi-Ardabili et al. (2021); Ricciardi et al. (2022); Wang et al. (2022); Ye et al. (2022); Zare Sakhvidi, Knobel, et al. (2022a); Y. D. Zhang et al. (2024b); Zhao et al. (2022); Zhao et al. (2021)
Proximity to greenspace	16 (35.6%)	Bianconi et al. (2023); De la Fuente et al. (2021); Li et al. (2023); Masdor et al. (2023); Patwary et al. (2024); Rahimi-Ardabili et al. (2021); Ricciardi et al. (2022); Rojas-Rueda et al. (2021); Wang et al. (2022); Ye et al. (2022); Yuan et al. (2021); Zare Sakhvidi, Knobel, et al. (2022a); Zare Sakhvidi, Yang, et al. (2022b); Zhang et al. (2021); Zhao et al. (2022); Zhao et al. (2021)
Green land cover	15 (33.3%)	Batterham et al. (2022); Bolanis et al. (2024); Browning et al. (2022); Cao et al. (2023); Gao et al. (2024); Rahimi-Ardabili et al. (2021); Ricciardi et al. (2022); Rigolon et al. (2021); Rojas-Rueda et al. (2021); Wang et al. (2022); Ye et al. (2022); Zagnoli et al. (2022); Zare Sakhvidi, Yang, et al. (2022b); Zhang et al. (2021); Zhao et al. (2022)
Tree canopy	10 (22.2%)	Ahmer et al. (2024); Bianconi et al. (2023); Bolanis et al. (2024); Gao et al. (2024); Patwary et al. (2024); Ricciardi et al. (2022); Wang et al. (2022); Yuan et al. (2021); Zagnoli et al. (2022); Zhao et al. (2022)
SAVI ^c^	8 (17.8%)	Aarthi et al. (2023); Bianconi et al. (2023); Patwary et al. (2024); Rahimi-Ardabili et al. (2021); Wang et al. (2022); Ye et al. (2022); Zare Sakhvidi, Knobel, et al. (2022a); Zhao et al. (2022)
EVI ^d^	8 (17.8%)	Cao et al. (2023); Ccami-Bernal et al. (2023); Patwary et al. (2024); Ricciardi et al. (2022); Wang et al. (2022); Ye et al. (2022); Y. D. Zhang et al. (2024b); Zhao et al. (2022)
Availability of greenspace	8 (17.8%)	Browning et al. (2022); Li and Lange (2023); Masdor et al. (2023); Rojas-Rueda et al. (2021); Yuan et al. (2021); X. Zhang et al. (2022c); Y. D. Zhang et al. (2024b); Zhao et al. (2022)
Gardening	7 (15.6%)	Briggs et al. (2022); Hsueh et al. (2022); Lampert et al. (2021); Rigolon et al. (2021); Tharrey and Darmon (2021); Tu (2022); Y. D. Zhang et al. (2024b)
Other ^a^	37 (82.2%)	Aarthi et al. (2023); Ahmer et al. (2024); Batterham et al. (2022); Bolanis et al. (2024); Browning et al. (2022); Cao et al. (2023); Ccami-Bernal et al. (2023); Chae et al. (2021); Chen et al. (2022); De la Fuente et al. (2021); Gao et al. (2024); Hsueh et al. (2022); Kang et al. (2022); Li and Lange (2023); Masdor et al. (2023); Patwary et al. (2024); Piva et al. (2024); Qiu et al. (2022); Rahimi-Ardabili et al. (2021); Ricciardi et al. (2022); Rigolon et al. (2021); Rojas-Rueda et al. (2021); Siah et al. (2023); Sivak et al. (2021); Wang et al. (2022); Wu et al. (2022); Ye et al. (2022); Yuan et al. (2021); Zagnoli et al. (2022); Zare Sakhvidi, Knobel, et al. (2022a); Zhang et al. (2021); X. Zhang et al. (2022c); Zhao et al. (2022); Zhao et al. (2021)

^a^ Other: proportion of greenspace, green exercise, frequency of greenspace exposure, viewing greenspace, density of greenspace, Light Detection and Ranging (LiDAR), greenspace exposure via Geographic Information System (GIS), land use, street view greenness, viewing greenspace photography/videography, Modified Soil Adjusted Vegetation Index (MSAVI), number of street trees, forest therapy, duration of greenspace exposure, forest bathing, Green View Index (GVI), greenspace ratio, number of green recreational facilities, Vegetative Continuous Field (VCF), greenness exposure by postcode, plant diversity, greenspace fragmentation, greenness satisfaction, greenspaces per inhabitant, greenspace area, quality of greenspace, intensity of exposure to nature, Leaf Area Index (LAI), total land area, type of greenspace, landscape elements, perceived quality and quantity of greenspace, activities in forest settings, loss of trees, biodiversity index, Moderate Resolution Imaging Spectroradiometer (MODIS), green patch area, green vision index, Vegetation Fractional Cover (VFC), green score, greening volunteerism, ecosystem count, nature meditation, nature-based programs, coordination of information of the environment (CORINE) land cover (CLC), undefined exposure to greenspace;

^b^ NDVI: Normalised Difference Vegetation Index;

^c^ SAVI: Soil-Adjusted Vegetation Index;

^d^ EVI: Enhanced Vegetation Index.

### Health outcomes

Health outcomes were sorted into the following categories: mental health and cognitive function, maternal health and birth outcomes, cardiovascular and metabolic outcomes, respiratory health and allergies, cancer, general health and QoL, and all-cause and cause-specific mortality. Health outcomes which did not align with these categories were classified as other health outcomes. Details of these categories and related ICD-10 codes are summarised in Table 4.

**Table 4.** Health outcomes investigated in the included systematic reviews.

Health outcome category	Health outcome	ICD-10 code(s) ^**a**^	Review articles	CCA ^**b**^
Mental health and cognitive function	Dementia	A50.4, B22.0, F00-F03, F84.3, G31.0	Zagnoli et al. (2022); Zhao et al. (2022)	3%
	Overall mental health/psychological outcomes	F00-F99	Batterham et al. (2022); Briggs et al. (2022); Hsueh et al. (2022); Kang et al. (2022); Lampert et al. (2021); Li and Lange (2023); Rahimi-Ardabili et al. (2021); Rojas-Rueda et al. (2021); Siah et al. (2023); Sivak et al. (2021); Tharrey and Darmon (2021); Tu (2022); Ye et al. (2022); Yi et al. (2022); Zhang et al. (2021)	1%
	Alzheimer’s dementia	F00	Zagnoli et al. (2022); Zhao et al. (2021)	3%
	Vascular dementia	F01	Zagnoli et al. (2022)	NA ^c^
	Non-Alzheimer’s dementia	B22.0, F01-F03, F10.7, F11.7, F12.7, F13.7, F14.7, F15.7, F16.7, F17.7, F18.7, F19.7, F84.3, G31.0	Zagnoli et al. (2022); Zhao et al. (2021)	3%
	Mood, affect	F06.3, F30-F39	Batterham et al. (2022); Briggs et al. (2022); Chen et al. (2022); Kang et al. (2022); Li and Lange (2023); Piva et al. (2024); Rahimi-Ardabili et al. (2021); Tharrey and Darmon (2021); Tu (2022); Yi et al. (2022)	1%
	Anxiety	F06.4, F34.1, F40-F41, F51.5, F93.0-F93.2, Z63.7	Batterham et al. (2022); Briggs et al. (2022); Hsueh et al. (2022); Kang et al. (2022); Lampert et al. (2021); Rahimi-Ardabili et al. (2021); Siah et al. (2023); Tu (2022); Ye et al. (2022); Yi et al. (2022); Zhang et al. (2021); X. Zhang et al. (2022c)	1%
	Schizophrenia	F20-F29	Batterham et al. (2022)	NA
	Depression	F32-F33	Batterham et al. (2022); Briggs et al. (2022); Chen et al. (2022); Hsueh et al. (2022); Kang et al. (2022); Lampert et al. (2021); Piva et al. (2024); Rahimi-Ardabili et al. (2021); Rojas-Rueda et al. (2021); Siah et al. (2023); Sivak et al. (2021); Tu (2022); Ye et al. (2022); Yi et al. (2022); Zhang et al. (2021)	1%
	Stress	F43, Z73	Briggs et al. (2022); Lampert et al. (2021); Li and Lange (2023); Piva et al. (2024); Rahimi-Ardabili et al. (2021); Siah et al. (2023); Sivak et al. (2021); Tharrey and Darmon (2021); Tu (2022); Ye et al. (2022)	1%
	Post traumatic stress disorder	F43.1	Briggs et al. (2022); Hsueh et al. (2022); Lampert et al. (2021); Tu (2022)	7%
	Autism	F84.0-F84.1	Ye et al. (2022)	NA
	Behavioural and psychological distress, emotional wellbeing, neurocognitive development	F90-F98	Chen et al. (2022); Tharrey and Darmon (2021); Zare Sakhvidi, Knobel, et al. (2022a); Zhang et al. (2021)	0%
	Attention deficit hyperactivity disorder	F90.0	Rahimi-Ardabili et al. (2021); Ye et al. (2022); Zare Sakhvidi, Knobel, et al. (2022a)	4%
	Alzheimer’s disease	G30	Zagnoli et al. (2022)	NA
	Cognition	R40-R44	Li and Lange (2023); Piva et al. (2024); Rahimi-Ardabili et al. (2021); Ricciardi et al. (2022); Rojas-Rueda et al. (2021); Siah et al. (2023); Tu (2022); Ye et al. (2022); Zagnoli et al. (2022); Zare Sakhvidi, Knobel, et al. (2022a); Zhao et al. (2022)	1%
	Suicidal ideation	R45.8	Bolanis et al. (2024)	NA
	Self-harm	X60-X84	Bolanis et al. (2024)	NA
	Neurochemical response	Z00.4	Piva et al. (2024)	NA
	Burnout	Z73.0	Briggs et al. (2022)	NA
Maternal health and birth outcomes	Overall birth and maternal health	P00-P96, O00-O99	Browning et al. (2022)	NA
	Birthweight	P07, P08	Ahmer et al. (2024); Rahimi-Ardabili et al. (2021)	0%
	Low birthweight	P07.0, P07.1	Ahmer et al. (2024)	NA
	Preterm birth	P07.2, P07.3	Ahmer et al. (2024); Rigolon et al. (2021)	15%
	Small for gestational age	P05.1	Ahmer et al. (2024)	NA
	Miscarriage	O03	Rahimi-Ardabili et al. (2021)	NA
	Life expectancy	NA	Rojas-Rueda et al. (2021)	NA
Cardiovascular and metabolic health	Diabetes	E10-E14, E23.2, O24	Aarthi et al. (2023); Browning et al. (2022); Ccami-Bernal et al. (2023); De la Fuente et al. (2021); Rahimi-Ardabili et al. (2021); Rigolon et al. (2021)	3%
	Blood glucose level	E10-E16, E23.2, O24, R73	Aarthi et al. (2023); Siah et al. (2023)	0%
	Metabolic health	E70-E90	Ye et al. (2022)	NA
	Cholesterol	E78	Siah et al. (2023)	NA
	Metabolic syndrome	E88.8	Patwary et al. (2024); Rahimi-Ardabili et al. (2021)	1%
	Cardiovascular health	I00-I99	Bianconi et al. (2023); Browning et al. (2022); Rahimi-Ardabili et al. (2021); Rigolon et al. (2021); Rojas-Rueda et al. (2021); Siah et al. (2023); Ye et al. (2022); Yuan et al. (2021)	2%
	Heart failure	I09.0, I09.9 I11.0, I13.0, I43.8, I50, I97.1	Bianconi et al. (2023)	NA
	Hypertension	I10-I15	Rahimi-Ardabili et al. (2021); Zhao et al. (2022)	3%
	Ischaemic heart disease	I20-I25	Bianconi et al. (2023); Yuan et al. (2021)	10%
	Acute myocardial infarction	I21-I22	Bianconi et al. (2023); Yuan et al. (2021)	10%
	Coronary heart disease	I25.9	Bianconi et al. (2023); Rahimi-Ardabili et al. (2021); Yuan et al. (2021)	2%
	Stroke	I64	Bianconi et al. (2023); Rahimi-Ardabili et al. (2021); Yuan et al. (2021)	2%
	Heart rate	R00	Piva et al. (2024); Siah et al. (2023); Yi et al. (2022)	17%
	Blood pressure	Z01.3	Piva et al. (2024); Qiu et al. (2022); Rahimi-Ardabili et al. (2021); Siah et al. (2023); Sivak et al. (2021); Tharrey and Darmon (2021); Ye et al. (2022); Yi et al. (2022); Zhao et al. (2022)	2%
Respiratory health and allergies	Acute rhino conjunctivitis	H10	Wang et al. (2022)	NA
	Respiratory diseases	J00-J99	Browning et al. (2022); Rahimi-Ardabili et al. (2021); Rojas-Rueda et al. (2021)	0%
	Rhinitis	J00, J30.0, J31.0	Rahimi-Ardabili et al. (2021); Ye et al. (2022)	3%
	Pneumonia	J14-J18, J69, J84.9, P23, P24.9	Ye et al. (2022)	NA
	Bronchitis	J20, J40-J42, J44, J45.0, J45.9	Ye et al. (2022)	NA
	Allergic rhinitis	J30, J45.0	Cao et al. (2023); Tang et al. (2023); Wang et al. (2022); Wu et al. (2022); Ye et al. (2022)	9%
	Chronic obstructive pulmonary disorder	J44	Gao et al. (2024); Rahimi-Ardabili et al. (2021); Tang et al. (2023)	4%
	Asthma	J45-J46	Rahimi-Ardabili et al. (2021); Rigolon et al. (2021); Tang et al. (2023); Wang et al. (2022); Wu et al. (2022); Ye et al. (2022)	4%
	Eczema & dermatitis	L20-L30	Rahimi-Ardabili et al. (2021); Rigolon et al. (2021); Wang et al. (2022); Ye et al. (2022)	3%
	Food allergies	L27.2, L23.6, T78.0-T78.1	Wang et al. (2022); Ye et al. (2022)	9%
	Lung function	Z00.8	Piva et al. (2024); Tharrey and Darmon (2021); Ye et al. (2022)	0%
Cancer	All-site cancer	C00-D48	Browning et al. (2022); Li et al. (2023); Zare Sakhvidi, Yang, et al. (2022b)	5%
	Non-melanoma cancer	C00-C41, C45-D02, D04-D09	Zare Sakhvidi, Yang, et al. (2022b)	NA
	Mouth cancer	C00-C09, D00.0	Li et al. (2023); Zare Sakhvidi, Yang, et al. (2022b)	14%
	Throat cancer	C09-C13, C14.0, D00.0	Zare Sakhvidi, Yang, et al. (2022b)	NA
	Colorectal cancer	C19, C78.5, D01.1	Li et al. (2023); Masdor et al. (2023); Zare Sakhvidi, Yang, et al. (2022b)	10%
	Lung cancer	C34, C78.0, D02.2	Li et al. (2023); Rahimi-Ardabili et al. (2021); Tang et al. (2023); Zare Sakhvidi, Yang, et al. (2022b)	4%
	Skin cancer	C43-C44, C79.2, D03-D04	Li et al. (2023); Zare Sakhvidi, Yang, et al. (2022b)	14%
	Breast cancer	C50, D24, D05	Li et al. (2023); Zare Sakhvidi, Yang, et al. (2022b)	14%
	Prostate cancer	C61, D07.5	Li et al. (2023); Rigolon et al. (2021); Zare Sakhvidi, Yang, et al. (2022b)	3%
	Bladder cancer	C67, C79.1, D09.0	Li et al. (2023)	NA
	Brain tumour	C71, D33, C79.3, D43.0-D43.2	Zare Sakhvidi, Yang, et al. (2022b)	NA
	Leukaemia	C90.1, C91-C95, D47.1, D47.5	Ye et al. (2022)	NA
General health and quality of life	Overall/general health/physiological outcomes	A00-Y98	Lampert et al. (2021); Li and Lange (2023); Rahimi-Ardabili et al. (2021); Rigolon et al. (2021); Rojas-Rueda et al. (2021); Siah et al. (2023); Sivak et al. (2021); Tharrey and Darmon (2021); Ye et al. (2022); Yi et al. (2022)	1%
	Chronic diseases	A00-Q99	Lampert et al. (2021); Rahimi-Ardabili et al. (2021)	0%
	Pain	G50.1, H57.1, I20.9, K14.6, M25.5, M54.5, M54.6, M79.6, N94, R07, R10, R14, R30, R51-R52	Siah et al. (2023)	NA
	Fatigue	R53	Rahimi-Ardabili et al. (2021); Siah et al. (2023)	3%
	Quality of life	NA	Briggs et al. (2022); Lampert et al. (2021); Piva et al. (2024); Rahimi-Ardabili et al. (2021); Rojas-Rueda et al. (2021); Siah et al. (2023); Ye et al. (2022); Yi et al. (2022)	1%
All-cause and cause specific mortality	All-cause mortality	A00-V98	Browning et al. (2022); Rahimi-Ardabili et al. (2021); Rigolon et al. (2021); Yuan et al. (2021)	6%
	Non-accidental mortality	A00-T98, X60-Y09, Y35-Y89	Yuan et al. (2021)	NA
	Infectious & parasitic disease mortality	A00-B99	Yuan et al. (2021)	NA
	Dementia mortality	A50.4, B22.0, F00-F03, F84.3, G31.0	Zagnoli et al. (2022)	NA
	All-site cancer mortality	C00-D48	Li et al. (2023); Yuan et al. (2021); Zare Sakhvidi, Yang, et al. (2022b)	8%
	Oesophageal cancer mortality	C15, D00.1	Zare Sakhvidi, Yang, et al. (2022b)	NA
	Colorectal cancer mortality	C19, D01.1	Masdor et al. (2023)	NA
	Lung cancer mortality	C34, C78.0	Li et al. (2023); Tang et al. (2023); Zare Sakhvidi, Yang, et al. (2022b)	12%
	Prostate cancer mortality	C61	Masdor et al. (2023); Zare Sakhvidi, Yang, et al. (2022b)	5%
	Neurogenerative disease mortality	F00, G10-G37, G60-G99, R27.0, R27.8	Yuan et al. (2021); Zagnoli et al. (2022)	5%
	Circulatory mortality	I00-I99	Yuan et al. (2021)	NA
	Ischaemic heart disease mortality	I20-I25	Bianconi et al. (2023); Yuan et al. (2021)	10%
	Acute myocardial infarction	I21-I22	Yuan et al. (2021)	NA
	Cardiovascular disease mortality	I51.6	Bianconi et al. (2023); Yuan et al. (2021)	10%
	Stroke mortality	I64	Bianconi et al. (2023); Yuan et al. (2021)	10%
	Respiratory disease mortality	J00-J99	Yuan et al. (2021)	NA
	COPD mortality	J44	Tang et al. (2023)	NA
	Infant/neonatal mortality	P00-P04, P07-P08, P95	Rigolon et al. (2021); Rojas-Rueda et al. (2021)	1%
	Suicide mortality	T76, Z60-X84	Bolanis et al. (2024)	NA
Other health outcomes	Foodborne pathogens	A02, A04.7, A05, A32	Sivak et al. (2021)	NA
	Dysentery	A07.9, A09.0	Rahimi-Ardabili et al. (2021)	NA
	Tuberculosis	A15-A19	Rahimi-Ardabili et al. (2021)	NA
	West Nile virus	A92.3	Sivak et al. (2021)	NA
	Dengue	A97	Rahimi-Ardabili et al. (2021)	NA
	Dermatophytosis	B35	Sivak et al. (2021)	NA
	Malaria	B50-B54	Rahimi-Ardabili et al. (2021)	NA
	Intestinal parasites	B65-B83	Ye et al. (2022)	NA
	Toxocariasis	B83.0	Sivak et al. (2021)	NA
	Cortisol level	E24, E27.1-E27.2, E27.8, E35.1	Piva et al. (2024); Qiu et al. (2022); Siah et al. (2023)	6%
	Parkinson’s disease	G20	Zagnoli et al. (2022)	NA
	Multiple sclerosis	G35	Zagnoli et al. (2022)	NA
	Myopia	H44.2, H52.1	Ye et al. (2022)	NA
	Astigmatism	H52.2	Ye et al. (2022)	NA
	Irritable bowel disease	K51, K63.8	Ye et al. (2022)	NA
	Heavy metal contamination	R82.6, R78.7, T56, X49, Y19	Sivak et al. (2021)	NA
	Microbiota (gut, skin, oral, nasal)	Z01.8	Y. D. Zhang et al. (2024b)	NA
	Immune markers	Z01.8	Chae et al. (2021)	NA

^a^ ICD-10: International Classification of Diseases;

^b^ CCA: Corrected Coverage Area;

^c^ NA: Not applicable.

***Mental health and cognitive function***

A total of 24 systematic reviews examined the association between greenspace exposure and mental health or cognitive function, with only a slight overlap in original studies (CCA = 1%). Overall mental health and well-being were investigated in 11 systematic reviews ( Batterham et al., 2022; Chen et al., 2022; Hsueh et al., 2022; Kang et al., 2022; Lampert et al., 2021; Rahimi-Ardabili et al., 2021; Tharrey & Darmon, 2021; Tu, 2022; Ye et al., 2022; Zare Sakhvidi, Knobel, et al., 2022a; Zhang et al., 2021). Greenspace interventions, such as greening volunteerism ( Chen et al., 2022), collective gardening ( Tharrey & Darmon, 2021), community gardening ( Lampert et al., 2021), and horticultural therapy ( Tu, 2022), showed positive associations with mental health and wellbeing. Forest-based interventions (encompassing forest therapy, forest bathing, forest videos, and forest-walking programs) ( Kang et al., 2022) and overall greenspace exposure (encompassing area of greenspace, area of parks, and green coverage ratio) ( Rahimi-Ardabili et al., 2021) were linked to better mental health, including in children ( Ye et al., 2022). Mediating factors between greenspace and mental health, such as air quality, perceived stress, and physical activity, were also identified ( Zhang et al., 2021). However, some reviews reported mixed evidence on the relationship between greenspace exposure and overall mental health and wellbeing ( Batterham et al., 2022; Hsueh et al., 2022; Zare Sakhvidi, Knobel, et al., 2022a).

Depression was assessed in eight reviews ( Batterham et al., 2022; Briggs et al., 2022; Kang et al., 2022; Piva et al., 2024; Siah et al., 2023; Sivak et al., 2021; Yi et al., 2022; Y. Zhang et al., 2024a). Meta-analyses demonstrated protective associations for group-based gardening ( Briggs et al., 2022) and NDVI ( Y. Zhang et al., 2024a), though heterogeneity was a limitation. Beneficial associations were also reported for forest-based interventions ( Kang et al., 2022; Piva et al., 2024; Siah et al., 2023; Yi et al., 2022), but narrative synthesis yielded mixed results for general greenspace ( Batterham et al., 2022; Briggs et al., 2022) and vacant lots ( Sivak et al., 2021).

Anxiety outcomes appeared in five reviews ( Briggs et al., 2022; Kang et al., 2022; Siah et al., 2023; X. Zhang et al., 2022c; Y. Zhang et al., 2024a). Increased greenspace exposure ( X. Zhang et al., 2022c) and forest-based interventions ( Kang et al., 2022; Siah et al., 2023) were associated with reduced anxiety levels. Increased greenspace, specifically NDVI, was also associated with lower odds of anxiety ( Y. Zhang et al., 2024a). However, evidence for group-based gardening was inconsistent in narrative synthesis and no association was found in the meta-analysis ( Briggs et al., 2022).

Cognitive function was assessed in six reviews ( Rahimi-Ardabili et al., 2021; Ricciardi et al., 2022; Rojas-Rueda et al., 2021; Zagnoli et al., 2022; Y. Zhang et al., 2024a; Zhao et al., 2021). Meta-analyses concluded that higher greenspace was associated with a reduced risk of combined cognitive impairment and dementia ( Zhao et al., 2021) and increased NDVI was associated with a decreased odds of dementia ( Y. Zhang et al., 2024a). Conversely, Zagnoli et al. (2022) reported that increased greenspace was associated with an increased risk of cognitive impairment, though this finding was limited by the small number of original studies. Inconsistent ( Ricciardi et al., 2022), insufficient ( Rojas-Rueda et al., 2021), or no evidence ( Rahimi-Ardabili et al., 2021) was noted in other reviews.

Other mental and cognitive health outcomes were also explored ( Bolanis et al., 2024; Briggs et al., 2022; Kang et al., 2022; Li & Lange, 2023; Piva et al., 2024; Rojas-Rueda et al., 2021; Siah et al., 2023; Sivak et al., 2021; Zare Sakhvidi, Knobel, et al., 2022a; Y. Zhang et al., 2024a). Increased NDVI within 400 m (OR [odds ratio] = 0.59, 95%CI: 0.35, 0.99) and 1500 m (OR = 0.52, 95%CI: 0.30, 0.91) of residences was associated with reduced odds of non-psychotic mental disorders ( Rojas-Rueda et al., 2021). Higher NDVI was also linked to lower odds of schizophrenia and attention deficit hyperactivity disorder (ADHD) (Y. Zhang et al., 2024a). Green vacant lots ( Sivak et al., 2021) and passive or light physical activity in urban greenspaces ( Li & Lange, 2023) were associated with reduced psychological stress, while forest-based interventions were associated with emotions ( Kang et al., 2022) and mood ( Piva et al., 2024; Siah et al., 2023). Protective associations were suggested for self-harm and suicidal ideation ( Bolanis et al., 2024), but evidence was mixed for conduct problems and internalising/externalising disorders ( Zare Sakhvidi, Knobel, et al., 2022a). Overall, evidence on the association between greenspace and mental health was positive, while cognitive outcomes showed more inconsistent results. Most of this evidence, however, had a high risk of bias (n = 21, 87.5%; Supplementary Figure S1a) and critically low methodological quality (n = 21, 87.5%; Supplementary Figure S2a).

***Maternal health and birth outcomes***

Only five reviews investigated maternal health and birth outcomes ( Ahmer et al., 2024; Browning et al., 2022; Rahimi-Ardabili et al., 2021; Rigolon et al., 2021; Rojas-Rueda et al., 2021). These systematic reviews had greater overlap in original studies than other outcome categories (CCA = 5%). The evidence on birth outcomes was notably limited, as highlighted by Rojas-Rueda et al. (2021) and Rahimi-Ardabili et al. (2021), who only found one original study each. A meta-analysis showed that increased residential greenspace within a 250 m radius was associated with higher birthweight (BW) and reduced odds of low birthweight (LBW) ( Ahmer et al., 2024). Narrative synthesis also suggested that residential greenspace may be associated with reduced preterm birth (PTB) and small-for-gestational-age (SGA) births ( Ahmer et al., 2024). Urban settings enhanced the protective association between greenspace and birth outcomes ( Browning et al., 2022). There was no evidence that socioeconomic status (SES) was an effect modifier ( Rigolon et al., 2021). Overall, evidence on maternal health and birth outcomes was limited and inconclusive. Most of this evidence also had a high risk of bias (n = 4, 80.0%; Supplementary Figure S1b) and all studies had critically low methodological quality (n = 5, 100.0%; Supplementary Figure S2b).

***Cardiovascular and metabolic outcomes***

Cardiovascular and metabolic outcomes were examined in 17 reviews ( Aarthi et al., 2023; Bianconi et al., 2023; Browning et al., 2022; Ccami-Bernal et al., 2023; De la Fuente et al., 2021; Patwary et al., 2024; Piva et al., 2024; Qiu et al., 2022; Rahimi-Ardabili et al., 2021; Rigolon et al., 2021; Rojas-Rueda et al., 2021; Siah et al., 2023; Tharrey & Darmon, 2021; Ye et al., 2022; Yi et al., 2022; Yuan et al., 2021; Zhao et al., 2022), which had a slight overlap (CCA = 1%). Higher greenspace exposure was associated with a lower incidence of diabetes mellitus ( Ccami-Bernal et al., 2023; Rahimi-Ardabili et al., 2021) and reduced risk of type 2 diabetes mellitus (T2DM) ( De la Fuente et al., 2021). However, Aarthi et al. (2023) reported inconsistent evidence for this association. Greenspace exposure also showed inverse relationships with CVD, ischaemic heart disease (IHD), acute myocardial infarction (AMI), and stroke morbidity, except when greenspace was measured via tree canopy exposure ( Bianconi et al., 2023).

Forest-based interventions were beneficially associated with systolic blood pressure (SBP) and diastolic blood pressure (DBP) in some reviews ( Piva et al., 2024; Qiu et al., 2022; Zhao et al., 2022) while others found no notable effects ( Siah et al., 2023; Yi et al., 2022). Zhao et al. (2022) reported associations between NDVI and blood pressure (BP)/hypertension at buffers of 100m (OR = 0.91, 95%CI: 0.86, 0.97), 250-300m (OR = 0.98, 95%CI: 0.97, 0.99), 500m (OR = 0.94, 95%CI: 0.94, 0.95), and 1000m (OR = 0.98, 95%CI: 0.97, 0.99), but other systematic reviews reported inconsistent ( Ye et al., 2022) or inadequate evidence ( Tharrey & Darmon, 2021) on BP outcomes.

An increase in NDVI within a 500 m buffer was associated with lower odds of metabolic syndrome (MetS) ( Patwary et al., 2024). Heart rate (HR) showed minimal ( Piva et al., 2024) or no change ( Rahimi-Ardabili et al., 2021; Siah et al., 2023) in response to greenspace exposure. Greenspace exposure was linked to reduced CVD risk, but evidence on stroke and myocardial infarction remained inconsistent ( Yuan et al., 2021). Evidence indicated that beneficial associations between greenspace and cardiovascular outcomes were stronger in urban areas than in less urban areas ( Browning et al., 2022). Benefits were also larger for individuals with lower SES compared to those with higher SES ( Rigolon et al., 2021). While some systematic reviews on cardiovascular and metabolic outcomes reported inconsistent associations, most found beneficial results. However, most of these systematic reviews had a high risk of bias (n = 16, 88.9%; Supplementary Figure S1c) and critically low methodological quality (n = 15, 83.3%; Supplementary Figure S2c).

***Respiratory health and allergies***

A total of 12 reviews addressed respiratory health and allergies ( Browning et al., 2022; Cao et al., 2023; Gao et al., 2024; Piva et al., 2024; Rahimi-Ardabili et al., 2021; Rigolon et al., 2021; Rojas-Rueda et al., 2021; Tang et al., 2023; Tharrey & Darmon, 2021; Wang et al., 2022; Wu et al., 2022; Ye et al., 2022), with a slight overlap in original studies (CCA = 3%). Higher residential greenness at birth was associated with lower odds of allergic rhinitis (AR) in childhood (OR = 0.83, 95%CI: 0.72, 0.96) ( Wang et al., 2022) and lower odds of current asthma when measured within a 500 m buffer ( Wang et al., 2022). Similarly, a 0.1-unit increase in NDVI was associated with a reduced risk of asthma incidence (RR [relative risk] = 0.92, 95%CI: 0.85, 0.98) ( Tang et al., 2023). However, other reviews found no associations between greenspace exposure and AR ( Cao et al., 2023; Wu et al., 2022; Ye et al., 2022) or asthma ( Wu et al., 2022; Ye et al., 2022).

Greenspace exposure demonstrated beneficial associations with lung function in children ( Ye et al., 2022) and in individuals with chronic obstructive pulmonary disease (COPD) ( Gao et al., 2024). Mixed findings were reported for overall respiratory outcomes ( Rahimi-Ardabili et al., 2021), atopic dermatitis ( Wang et al., 2022), and food allergies ( Wang et al., 2022). Inadequate evidence was noted in other reviews ( Piva et al., 2024; Rojas-Rueda et al., 2021; Tharrey & Darmon, 2021). Urbanicity and SES did not modify the association between greenspace and respiratory health and atopic diseases ( Browning et al., 2022; Rigolon et al., 2021). Altogether, evidence on respiratory health and allergies was mixed. For most reviews, risk of bias was high (n = 9, 75%; Supplementary Figure S1d) and methodological quality was critically low (n = 11, 91.7%; Supplementary Figure S2d).

***Cancer***

The association between greenspace exposure and cancer was explored in eight reviews ( Browning et al., 2022; Li et al., 2023; Masdor et al., 2023; Rahimi-Ardabili et al., 2021; Rigolon et al., 2021; Tang et al., 2023; Ye et al., 2022; Zare Sakhvidi, Yang, et al., 2022b), which had a slight overlap (CCA = 3%). Tang et al. (2023) reported that a 0.1-unit increase in NDVI was associated with a reduced risk of lung cancer incidence, although high heterogeneity was observed across studies.

Insufficient evidence was found for an association between greenspace exposure and colorectal cancer ( Li et al., 2023; Masdor et al., 2023), lung cancer ( Rahimi-Ardabili et al., 2021; Zare Sakhvidi, Yang, et al., 2022b), breast cancer ( Zare Sakhvidi, Yang, et al., 2022b), prostate cancer ( Zare Sakhvidi, Yang, et al., 2022b), and all-site cancer ( Li et al., 2023) based on both quantitative and qualitative syntheses. Li et al. (2023) highlighted inconsistent findings for prostate, lung, breast, bladder, and skin cancers, further underscoring the need for more robust and comprehensive research to clarify the role of greenspace exposure in cancer outcomes. Other reviews were limited by insufficient evidence ( Browning et al., 2022; Rigolon et al., 2021; Ye et al., 2022). For the most part, mixed findings or no association were reported among systematic reviews on the association between greenspace and cancer. Most of these systematic reviews also had a high risk of bias (n = 6, 75.0%; Supplementary Figure S1e) and critically low methodological quality (n = 6, 75.0%; Supplementary Figure S2e).

***General health and quality of life***

General health and QoL were assessed in 12 reviews ( Briggs et al., 2022; Lampert et al., 2021; Li & Lange, 2023; Piva et al., 2024; Rahimi-Ardabili et al., 2021; Rigolon et al., 2021; Rojas-Rueda et al., 2021; Siah et al., 2023; Sivak et al., 2021; Tharrey & Darmon, 2021; Ye et al., 2022; Yi et al., 2022). These systematic reviews had a slight overlap in original studies (CCA = 1%). Forest bathing was linked to enhanced QoL ( Siah et al., 2023), while forest therapy demonstrated associations with improved physiological parameters ( Piva et al., 2024). Visits to parks with a companion (PR [prevalence ratio] = 1.12, 95% CI: 1.01–1.25) and the condition of trees in parks (PR = 1.20, 95% CI: 1.07–1.34) were also positively associated with QoL ( Rojas-Rueda et al., 2021). However, evidence regarding forest therapy and QoL was mixed ( Yi et al., 2022).

Li and Lange (2023) found that passive exposure to greenspace or light physical activity in green settings reduced physical stress. Gardening interventions were associated with better physical health ( Lampert et al., 2021) and wellbeing (SMD [standardised mean difference] = 0.37, 95% CI: 0.01, 0.73) ( Briggs et al., 2022). However, evidence on gardening and physiological health was mixed ( Tharrey & Darmon, 2021), and no association was observed with health-related QoL ( Briggs et al., 2022). In narrative synthesis, greenspace exposure was linked to improved general and physical health but not frailty, disability, or chronic diseases ( Rahimi-Ardabili et al., 2021). Rigolon et al. (2021) identified SES as an important modifier in the relationship between greenspace and general health. Evidence on the risk of physical injury ( Sivak et al., 2021), hospital admission ( Ye et al., 2022), and sleep quality/sufficiency ( Ye et al., 2022) was limited. Overall, systematic reviews on general health and QoL showed protective associations with greenspace exposure but had a high risk of bias (n = 12, 100.0%; Supplementary Figure S1f ) and critically low methodological quality (n = 12, 100.0%; Supplementary Figure S2f ).

***All-cause and cause-specific mortality***

All-cause and cause-specific mortality was examined in 12 reviews ( Bianconi et al., 2023; Bolanis et al., 2024; Browning et al., 2022; Li et al., 2023; Masdor et al., 2023; Rahimi-Ardabili et al., 2021; Rigolon et al., 2021; Rojas-Rueda et al., 2021; Tang et al., 2023; Yuan et al., 2021; Zagnoli et al., 2022; Zare Sakhvidi, Yang, et al., 2022b), with a slight overlap between reviews (CCA = 2%). Incremental increases in NDVI were associated with reduced mortality risks from lung cancer ( Li et al., 2023), prostate cancer ( Li et al., 2023), and COPD ( Tang et al., 2023). Bianconi et al. (2023) reported that urban greenspace was associated with a reduced risk of CVD, IHD, and cerebrovascular disease (CBVD) mortality. Greenspace also showed a favourable association with childhood mortality ( Rojas-Rueda et al., 2021). The protective association between greenspace and suicide mortality was stronger in females than males ( Bolanis et al., 2024), and the association with all-cause mortality was stronger in urban than less urban areas ( Browning et al., 2022).

Inconsistent or insufficient evidence was reported for associations with mortality due to prostate cancer ( Li et al., 2023; Masdor et al., 2023), lung cancer ( Tang et al., 2023), oesophageal cancer ( Zare Sakhvidi, Yang, et al., 2022b), all-cause cancer ( Yuan et al., 2021), stroke ( Yuan et al., 2021), dementia ( Zagnoli et al., 2022), and all-cause mortality ( Rahimi-Ardabili et al., 2021). Overall, evidence on all-cause and cause-specific mortality was inconsistent and tended to have a high risk of bias (n = 9, 75.0%; Supplementary Figure S1g) and critically low methodological quality (n = 9, 75.0%; Supplementary Figure S2g).

***Other health outcomes***

A total of seven reviews explored health outcomes which did not fit the above categories ( Chae et al., 2021; Piva et al., 2024; Qiu et al., 2022; Rahimi-Ardabili et al., 2021; Sivak et al., 2021; Ye et al., 2022; Y. D. Zhang et al., 2024b). These reviews had only a slight overlap in original studies (CCA = 1%), but were all rated as having a high risk of bias (n = 7, 100.0%; Supplementary Figure S1h) and critically low methodological quality (n = 7, 100.0%; Supplementary Figure S2h). Forest therapy was associated with an increase in natural killer (NK) cell counts and activity, as well as changes in cytotoxic effector molecules and proinflammatory cytokines ( Chae et al., 2021). Decreased salivary cortisol concentration (SCC) was observed in participants who underwent forest therapy compared to urban controls (MD [mean difference] = −0.07, 95%CI: −0.10, −0.04), though high heterogeneity (I ^2^ = 83.9%) was noted ( Qiu et al., 2022). Walking in forests was associated with improved neurochemical markers, including reductions in interleukin-6 (IL-6) and tumour necrosis factor alpha (TNF-α) levels ( Piva et al., 2024).

Rahimi-Ardabili et al. (2021) linked greenspace exposure to a lower incidence of dysentery but an increased incidence of malaria, dengue fever, and tuberculosis in China. However, these conclusions were constrained by limited original studies. Protective associations were suggested for infections, myopia, and astigmatism, though insufficient evidence precluded definitive conclusions ( Ye et al., 2022).

Vacant lots with greenspace were associated with elevated blood lead levels in children and increased rates of mosquitoes carrying West Nile Virus ( Sivak et al., 2021). Connections between vacant lots and dermatophytosis and toxocariasis were also indicated ( Sivak et al., 2021). Y. D. Zhang et al. (2024b) reported that greenspace exposure was positively associated with the diversity and composition of gut and skin microbiota. It was also linked to an increase in probiotics and a reduction in pathogens, but evidence for nasal, ocular, and oral microbiota was inconclusive ( Y. D. Zhang et al., 2024b).

### Quantitative data summary

While this review did not conduct quantitative synthesis, Table 5 provides a comprehensive list of the quantitative measures reported across the systematic reviews. Effect estimates based on more than one original study were listed in this table. 

**Table 5.** Listing of quantitative data from included reviews' meta-analyses.

Outcome	Review article	No. & type of original studies included	Exposure measure	Effect measure	Estimate (95%CI ^a^) from meta-analysis
Mental health and cognitive function					
Dementia	Zagnoli et al. (2022)	6 (2 cross-sectional, 2 cohort, 1 combined cohort case-control)	NDVI ^b^	RR ^c^ (highest vs lowest greenness categories, 1-unit increases)	0.98 (0.90, 1.06)
		6 (2 cross-sectional, 2 cohort, 1 case-control, 1 ecological	Land use/land change		0.99 (0.93, 1.05)
	Y. Zhang et al. (2024a)	8 (cross-sectional)	Any greenspace exposure	OR ^d^ (dose-response not reported)	0.95 (0.93, 0.96)
		5 (cohort)	NDVI		0.95 (0.94, 0.96)
		3 (cohort)	Area of greenspace		0.93 (0.84, 1.03)
Dementia/cognitive impairment	Zhao et al. (2021)	8 (4 cohort, 2 cross-sectional, 2 case-control)	Residential greenness	OR (dose-response not reported)	0.97 (0.95, 1.00)
		4 (cohort)			0.96 (0.95, 0.98)
Cognitive impairment	Zagnoli et al. (2022)	2 (cross-sectional)	Land use/land change	RR (highest vs lowest greenness categories)	1.47 (1.22, 1.76)
Depression	Briggs et al. (2022)	8 (RCT)	Gardening	SMD ^e^ (intervention vs control)	-0.43 (-0.79, -0.06)
	Kang et al. (2022)	5 (RCT)	Forest therapy	SMD (pre- vs post-intervention)	1.36 (0.55, 2.17)
	Siah et al. (2023)	10 (5 quasi-experimental, 4 RCT, 1 crossover design)	Forest bathing	SMD (intervention vs control)	-0.67 (-0.99, -0.35)
	Yi et al. (2022)	4 (RCT)	Forest therapy	SMD (intervention vs control)	-1.46 (-2.80, -0.12)
	Y. Zhang et al. (2024a)	37 (29 cross-sectional, 8 cohort)	Any greenspace exposure Any greenspace exposure	OR (dose-response not reported)	0.89 (0.86, 0.93)
		35 (28 cross-sectional, 7 cohort)			0.96 (0.94, 0.97)
		5 (cross-sectional)	View of greenery		0.89 (0.74, 1.06)
		16 (12 cross-sectional, 4 cohort)	NDVI		0.95 (0.91, 0.98)
		19 (16 cross-sectional, 3 cohort)	Area of greenspace		0.97 (0.94, 1.01)
		3 (cross-sectional)	Green space accessibility		0.85 (0.67, 1.08)
		3 (2 cross-sectional, 1 cohort)	Parks		1.00 (0.99, 1.00)
		4 (2 cohort, 2 cross-sectional)	Other greenspace exposures ^f^		0.88 (0.83, 0.94)
Anxiety	Briggs et al. (2022)	5 (RCT)	Gardening	SMD (intervention vs control)	-0.42 (-1.00, 0.16)
	Kang et al. (2022)	5 (RCT)	Forest therapy	SMD (pre- vs post-intervention)	0.88 (0.18, 1.58)
	Siah et al. (2023)	7 (4 RCT, 2 quasi-experimental, 1 crossover design)	Forest bathing	SMD (intervention vs control)	-0.84 (-1.42, -0.25)
	X. Zhang et al. (2022c)	24 (12 randomised crossover, 7 non-randomised crossover, 3 randomised parallel group, 1 2x2 factorial design, 1single group crossover)	Natural environment	SMD (pre- vs post-intervention)	-1.28 (-1.65, -0.92)
	Y. Zhang et al. (2024a)	14 (11 cross-sectional, 3 cohort)	Any greenspace exposure	OR (dose-response not reported)	0.94 (0.92, 0.96)
		9 (7 cross-sectional, 2 cohort)	Any greenspace exposure		0.94 (0.91, 0.97)
		3 (cross-sectional)	View of greenery		0.84 (0.78, 0.91)
		2 (cross-sectional)	Area of greenspace		0.98 (0.93, 1.03)
		3 (2 cross-sectional, 1 cohort)	NDVI		0.95 (0.92, 0.98)
		2 (1 cohort, 1 cross-sectional)	Other greenspace exposures ^g^		0.93 (0.85, 1.03)
Stress	Briggs et al. (2022)	3 (RCT)	Gardening	SMD (intervention vs control)	-0.17 (-0.68, 0.35)
ADHD ^h^	Y. Zhang et al. (2024a)	5 (3 cohort, 2 cross-sectional)	Any greenspace exposure	OR (dose-response not reported)	0.89 (0.86, 0.92)
Positive emotions	Kang et al. (2022)	4 (RCT)	Forest therapy	SMD (pre- vs post-intervention)	0.91 (0.34, 1.47)
Negative emotions	Kang et al. (2022)	3 (RCT)	Forest therapy	SMD (pre- vs post-intervention)	1.37(0.81, 1.93)
Psychiatric disorders	Y. Zhang et al. (2024a)	60 (32 cross-sectional, 28 cohort)	Any greenspace exposure	OR (dose not reported)	0.91 (0.89, 0.92)
Schizophrenia	Y. Zhang et al. (2024a)	7 (cohort)	Any greenspace exposure	OR (dose-response not reported)	0.74 (0.67, 0.82)
		7 (cohort)	Any greenspace exposure		0.75 (0.69, 0.81)
		5 (cohort)	NDVI		0.72 (0.64, 0.82)
		4 (cohort)	Area of greenspace		0.77 (0.70, 0.85)
Overall mental health	Kang et al. (2022)	6 (RCT)	Forest therapy	SMD (pre- vs post-intervention)	1.25 (0.93, 1.57)
	Tu (2022)	18 (RCT)	Horticultural therapy	Hedge’s g (intervention vs control)	0.55 (0.38, 0.72)
Maternal health and birth outcomes					
Birthweight	Ahmer et al. (2024)	8 (7 cross-sectional, 1 case-control)	NDVI 250m buffer	beta standardised regression coefficient (per 0.1-unit change)	8.95 (1.63, 16.27)
		8 (7 cross-sectional, 1 case-control)	NDVI 250m buffer		9.86 (1.91, 17.81)
		11 (9 cross-sectional, 1 case-control, 1 cohort)	NDVI 500m buffer		12.83 (4.69, 20.97)
		13 (11 cross-sectional, 1 case-control, 1 cohort)	NDVI 250m & 500m buffer		11.18 (5.69, 16.67)
Low birth weight	Ahmer et al. (2024)	6 (5 cross-sectional, 1 cohort)	NDVI 250m buffer	OR (per 0.1-unit change)	0.97 (0.96, 0.98)
Cardiovascular and metabolic outcomes					
Systolic blood pressure	Qiu et al. (2022)	20 (10 parallel RCT, 8 crossover RCT, 2 non-RCT)	Forest therapy	MD ^i^ (change in intervention vs control)	-3.44 (-5.74, -1.14)
	Siah et al. (2023)	13 (9 RCT, 2 quasi-experimental, 1 non-randomised crossover, 1 crossover)	Forest bathing	MD (intervention vs control)	-1.66 (-4.30, 0.97)
	Yi et al. (2022)	6 (RCT)	Forest therapy	MD (intervention vs control)	-0.24 (-2.70, 2.23)
	Zhao et al. (2022)	5 (4 cross-sectional, 1 cohort)	NDVI 500m buffer	beta standardised regression coefficient (per 0.1-unit increase)	-0.77 (-1.23, -0.32)
Diastolic blood pressure	Qiu et al. (2022)	21 (10 parallel RCT, 9 crossover RCT, 2 non-RCT)	Forest therapy	MD (change in intervention vs control)	-3.07 (-5.59, -0.54)
	Siah et al. (2023)	13 (9 RCT, 2 quasi-experimental, 1 non-randomised crossover, 1 crossover)	Forest bathing	MD (intervention vs control)	-3.09 (-7.52, 1.34)
	Yi et al. (2022)	5 (RCT)	Forest therapy (post-intervention vs control)	MD (intervention vs control)	0.94 (-3.20, 5.07)
	Zhao et al. (2022)	5 (4 cross-sectional, 1 cohort)	NDVI 500m buffer	beta standardised regression coefficient (per 0.1-unit increase)	-0.32 (-0.57, -0.07)
Blood pressure/hypertension	Zhao et al. (2022)	3 (cross-sectional)	NDVI 100m buffer	OR (per 0.1-unit increase)	0.91 (0.86, 0.97)
		4 (cross-sectional)	NDVI 250/300m buffer		0.98 (0.97, 0.99)
		8 (7 cross-sectional, 1 case-control)	NDVI 500m buffer		0.94 (0.93, 0.95)
		4 (cross-sectional)	NDVI 1000m buffer		0.98 (0.97, 0.99)
		4 (3 cross-sectional, 1 longitudinal)	Proportion of greenspace	OR (per 1% increase)	0.99 (0.99, 1.00)
		3 (cross-sectional)	Distance to greenspace	OR (per 500m difference)	1.03 (0.96, 1.10)
Heart rate	Siah et al. (2023)	5 (3 RCT, 1 quasi-experimental, 1 crossover)	Forest bathing	MD (intervention vs control)	-0.42 (-3.32, 2.49)
Metabolic syndrome	Patwary et al. (2024)	4 (3 cohort, 1 cross-sectional)	NDVI 500m buffer	OR (dose-response not reported)	0.90 (0.87, 0.93)
Respiratory health and allergies					
Asthma	Tang et al. (2023)	9 (5 cross-sectional, 2 case-control, 2 cohort)	NDVI 200-300m buffer	RR (per 0.1-unit increase)	0.92 (0.86, 0.98)
		9 (6 cross-sectional, 2 case-control, 1 cohort)	NDVI 500m buffer		0.93 (0.85, 1.01)
		8 (5 cross-sectional, 1 case-control, 1 cohort, 1 longitudinal)	NDVI 1000m buffer		0.87 (0.81, 0.93)
	Ye et al. (2022)	10 (5 cohort, 5 cross-sectional)	NDVI	OR (per 0.1-unit increase)	0.94 (0.84, 1.05)
Current asthma	Wang et al. (2022)	3 (cross-sectional)	NDVI 100m buffer	OR (per IQR increase)	1.02 (0.90, 1.14)
		3 (cross-sectional)	NDVI 100-500m buffer		0.94 (0.78, 1.13)
		4 (cross-sectional)	NDVI 500m buffer		0.88 (0.78, 0.99)
		4 (cross-sectional)	NDVI 1000m buffer		0.94 (0.86, 1.02)
		4 (cross-sectional)	NDVI 100-1000m buffer		0.94 (0.88, 1.00)
	Wu et al. (2022)	3 (2 cross-sectional, 1 case-control)	NDVI >0-100m buffer	OR (per 0.1-unit increase)	0.98 (0.90, 1.07)
		4 (2 cross-sectional, 1 case-contol, 1 cohort)	NDVI >100-300m buffer		0.99 (0.91, 1.07)
		4 (2 cross-sectional, 1 case-contol, 1 cohort)	NDVI >300-500m buffer		1.00 (0.91, 1.09)
		4 (2 cross-sectional, 1 case-contol, 1 cohort)	NDVI >500-1000m buffer		0.98 (0.90, 1.08)
Ever had asthma	Wu et al. (2022)	4 (2 case-control, 2 cross-sectional)	NDVI >0-100m buffer	OR (per 0.1-unit increase)	1.04 (0.92, 1.16)
		4 (2 case-control, 2 cross-sectional)	NDVI >100-300m buffer		1.00 (0.99, 1.02)
		3 (2 cross-sectional, 1 case-control)	NDVI >300-500m buffer		1.04 (0.90, 1.22)
Asthma incidence	Tang et al. (2023)	9 (4 cross-sectional, 2 case-control, 2 cohort, 1 longitudinal)	NDVI	RR (per 0.1-unit increase)	0.92 (0.85, 0.98)
Asthma prevalence	Tang et al. (2023)	4 (2 cohort, 2 cross-sectional)	NDVI	RR (per 0.1-unit increase)	0.89 (0.74, 1.08)
COPD ^j^	Tang et al. (2023)	4 (3 cross-sectional, 1 cohort)	NDVI 500m buffer	RR (per 0.1-unit increase)	0.95 (0.89, 1.02)
		3 (2 cross-sectional, 1 cohort)	NDVI 1000m buffer		0.92 (0.83, 1.03)
COPD incidence	Tang et al. (2023)	2 (1 cohort, 1 cross-sectional)	NDVI	RR (per 0.1-unit increase)	0.92 (0.83, 1.03)
COPD prevalence	Tang et al. (2023)	2 (cross-sectional)	NDVI	RR (per 0.1-unit increase)	1.00 (0.90, 1.12)
Allergic rhinitis	Cao et al. (2023)	5 (3 cohort, 2 cross-sectional)	NDVI 100m buffer	OR (per 10% increase)	1.00 (1.00, 1.00)
		2 (1 cohort, 1 cross-sectional)	NDVI 200m buffer		1.00 (0.99, 1.01)
		3 (2 cohort, 1 cross-sectional)	NDVI 250m buffer		1.00 (1.00, 1.00)
		3 (cohort)	NDVI 300m buffer		1.00 (0.99, 1.02)
		5 (3 cohort, 2 cross-sectional)	NDVI 500m buffer		0.99 (0.97, 1.01)
		2 (1 cohort, 1 cross-sectional)	NDVI 1000m buffer		0.99 (0.97, 1.01)
		10 (7 cohort, 3 cross-sectional)	NDVI 100-1000m buffer		1.00 (0.99, 1.00)
	Tang et al. (2023)	5 (2 cohort, 2 cross-sectional, 2 case-control)	NDVI 200-300m buffer	RR (per 0.1-unit increase)	1.02 (0.95, 1.09)
		7 (3 cohort, 3 cross-sectional, 1 cohort)	NDVI 500m buffer		0.99 (0.94, 1.03)
		4 (2 cross-sectional, 1 cohort)	NDVI 1000m buffer		0.99 (0.91, 1.06)
	Wang et al. (2022)	2 (cohort)	NDVI 100m buffer	OR (3 ^rd^ tertile vs 1 ^st^ tertile)	0.65 (0.27, 1.55)
		3 (cohort)	NDVI 100-500m buffer		0.75 (0.59, 0.95)
		2 (cohort)	NDVI 500m buffer		0.91 (0.70, 1.19)
		4 (cohort)	NDVI		0.83 (0.72, 0.96)
	Wu et al. (2022)	3 (2 cross-sectional, 1 case-control)	NDVI 100m buffer	OR (per 0.1-unit increase)	0.98 (0.95, 1.02)
		4 (2 cross-sectional, 1 case-control, 1 cohort)	NDVI 500m buffer		0.99 (0.94, 1.04)
		3 (2 cross-sectional, 1 case-control)	NDVI 1000m buffer		1.00 (0.95, 1.05)
	Ye et al. (2022)	6 (3 cohort, 3 cross-sectional)	NDVI	OR (per 0.1-unit increase)	0.95 (0.85, 1.06)
Allergic rhinitis incidence	Tang et al. (2023)	6 (4 cohort, 1 case-control, 1 cross-sectional)	NDVI	RR (per 0.1-unit increase)	1.02 (0.97, 1.08)
Allergic rhinitis prevalence	Tang et al. (2023)	2 (cross-sectional)	NDVI	RR (per 0.1-unit increase)	0.91 (0.64, 0.95)
Cancer					
All-site cancer	Li et al. (2023)	6 (cohort)	NDVI	HR ^k^ (per 0.1-unit increase)	0.98 (0.95, 1.01)
Prostate cancer	Li et al. (2023)	4 (cohort)	NDVI	HR (per 0.1-unit increase)	0.95 (0.85, 1.05)
	Zare Sakhvidi, Yang, et al. (2022b)	2 (1 cohort, 1 case-control)	NDVI	OR/RR (per 0.1-unit increase)	0.91 (0.69, 1.20)
		2 (cohort)	NDVI	HR (per 0.1-unit increase)	0.98 (0.69, 1.40)
Colorectal cancer	Li et al. (2023)	2 (cohort)	NDVI	HR (per 0.1-unit increase)	1.00 (0.96, 1.04)
Bladder cancer	Li et al. (2023)	2 (cohort)	NDVI	HR (per 0.1-unit increase)	0.83 (0.45, 1.53)
Breast cancer	Li et al. (2023)	5 (cohort)	NDVI	HR (per 0.1-unit increase)	0.95 (0.90, 1.01)
	Zare Sakhvidi, Yang, et al. (2022b)	2 (1 cohort, 1 case-control)	NDVI	OR/RR (per 0.1-unit increase)	1.01 (0.80, 1.27)
		2 (cohort)	NDVI	HR (per 0.1-unit increase)	0.83 (0.47, 1.48)
Lung cancer	Li et al. (2023)	3 (cohort)	NDVI	HR (per 0.1-unit increase)	0.97 (0.95, 0.98)
	Tang et al. (2023)	5 (3 cohort, 1 case-control, 1 ecological)	NDVI	RR (per 0.1-unit increase)	0.62 (0.40, 0.95)
		3 (cohort)	NDVI 500m buffer		0.70 (0.46, 1.06)
		2 (1 cohort, 1 ecological)	NDVI 1000m buffer		0.20 (0.01, 4.48)
	Zare Sakhvidi, Yang, et al. (2022b)	4 (cross-sectional)	NDVI	IRR ^l^ (per 0.1-unit increase)	0.99 (0.85, 1.48)
		2 (cohort)	NDVI	HR (per 0.1-unit increase)	1.00 (0.87, 1.38)
		2 (1 case-control, 1 cross-sectional)	NDVI	OR/RR (per 0.1-unit increase)	1.00 (0.84, 1.20)
Skin cancer	Li et al. (2023)	2 (cohort)	NDVI	HR (per 0.1-unit increase)	0.86 (0.57, 1.30)
General health and quality of life					
Quality of life	Briggs et al. (2022)	7 (RCT)	Gardening	SMD (intervention vs control)	-0.06 (-0.45, 0.34)
Overall wellbeing	Briggs et al. (2022)	4 (RCT)	Gardening	SMD (intervention vs control)	0.37 (0.01, 0.73)
All-cause and cause-specific mortality					
All-cause mortality	Yuan et al. (2021)	8 (cohort)	NDVI	HR (per 0.1-unit increase)	0.99 (0.97, 1.00)
Cardiovascular mortality	Bianconi et al. (2023)	6 (cohort)	NDVI	HR (per IQR increase)	0.94 (0.91, 0.97)
	Yuan et al. (2021)	4 (cohort)	NDVI	HR (per 0.1-unit increase)	0.99 (0.89,1.09)
Ischaemic heart disease mortality	Bianconi et al. (2023)	5 (cohort)	NDVI	HR (per IQR increase)	0.96 (0.93, 0.99)
	Yuan et al. (2021)	3 (cohort)	NDVI	HR (per 0.1-unit increase)	0.96 (0.88, 1.05)
Stroke mortality	Yuan et al. (2021)	4 (cohort)	NDVI	HR (per 0.1-unit increase)	0.77 (0.59, 1.00)
Cerebrovascular disease mortality	Bianconi et al. (2023)	5 (cohort)	NDVI	HR (per IQR increase)	0.96 (0.94, 0.97)
Respiratory mortality	Yuan et al. (2021)	5 (cohort)	NDVI	HR (per 0.1-unit increase)	0.99 (0.89, 1.10)
COPD mortality	Tang et al. (2023)	3 (2 cohort, 1 ecological)	NDVI	RR (per 0.1-unit increase)	0.95 (0.92, 0.99)
		2 (1 cohort, 1 ecological)	NDVI 1000m buffer		0.93 (0.88, 0.98)
Lung cancer mortality	Li et al. (2023)	4 (cohort)	NDVI	HR (per 0.1-unit increase)	0.97 (0.95, 0.98)
	Tang et al. (2023)	6 (4 cohort, 2 ecological)	NDVI	RR (per 0.1-unit increase)	0.98 (0.96, 1.01)
		5 (4 cohort, 1 ecological)	NDVI 250-300m buffer		0.98 (0.94, 1.01)
		4 (cohort)	NDVI 1000m buffer		0.98 (0.94, 1.03)
Other health outcomes					
Salivary cortisol concentration	Qiu et al. (2022)	13 (10 crossover RCT, 3 non-RCT)	Forest therapy	MD (change in intervention vs control)	-0.07 (-0.10, -0.04)

^a^ 95% CI: 95% confidence interval;

^b^ NDVI: Normalised differential vegetation index;

^c^ RR: Risk ratio;

^d^ OR: Odds ratio;

^e^ SMD: Standardised mean difference;

^f^ Encompasses residential greenness and decreased visits to greenspace;

^g^ Encompasses number of neighbourhood parks, distance to useable green space, distance to total green space proportion of useable green space within 300m, proportion of total green space within 300m, proportion of useable green space within 3km, and proportion of total green space within 3km;

^h^ ADHD: Attention deficit hyperactivity disorder;

^i^ MD: Mean difference;

^j^ COPD: Chronic obstructive pulmonary disorder;

^k^ HR: Hazard ratio;

^l^ IRR: Incidence rate ratios.

### Quality of evidence and risk of bias

Of the 45 systematic reviews included, most exhibited a high risk of bias (n = 35, 77.8%) ( Figure 1; extended data: Supplementary Table S2) and critically low methodological quality (n = 36, 80.0%) ( Table 6; extended data: Supplementary Figure S3). Only one review was deemed high quality ( Patwary et al., 2024). The overall CCA was calculated as 1%, indicating only a slight overlap in the original studies.

**Figure 1.**
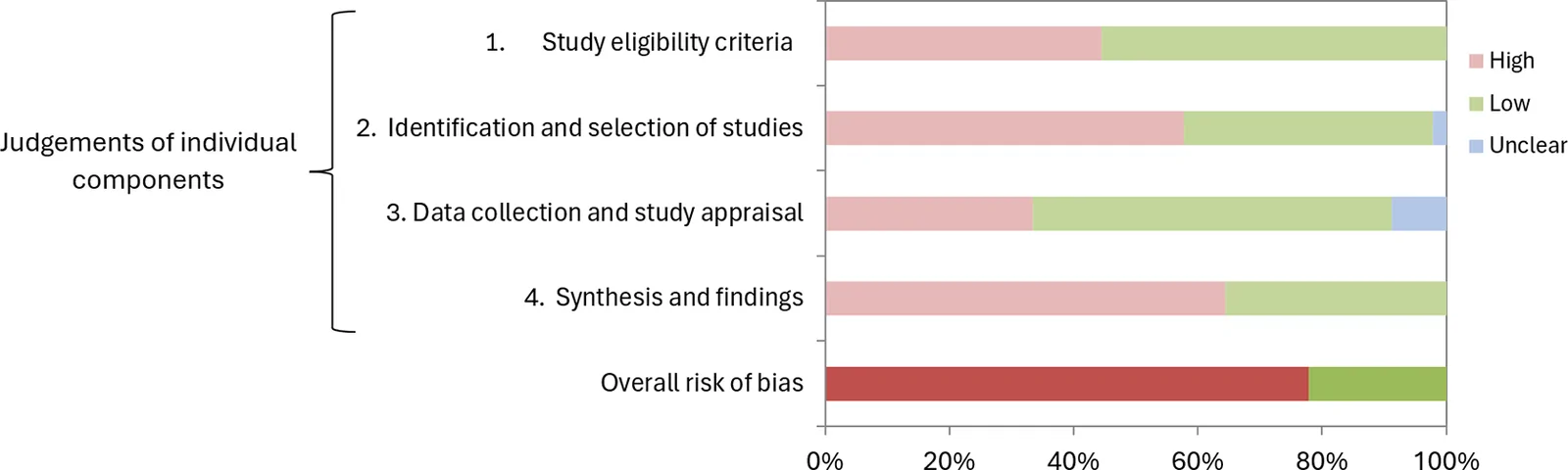
Risk of bias assessment of included reviews using ROBIS. Rows 1-4 show judgements on individual criteria while the bottom row shows the overall risk of bias.

**Table 6.** Methodological quality assessment of included systematic reviews using AMSTAR-2.

Question number ^a^	1	2	3	4	5	6	7	8	9	10	11	12	13	14	15	16	Quality
Author, Year																	
Aarthi et al. (2023)	Y ^b^	PY	N	PY	Y	N	N	PY	NA/PY	N	N/NA	N	Y	Y	N	Y	CL
Ahmer et al. (2024)	Y	Y	N	N	Y	Y	N	PY	NA/N	N	NA/Y	Y	Y	Y	Y	Y	CL
Batterham et al. (2022)	Y	N	N	PY	Y	Y	N	N	NA/N	N	NA/NA	NA	Y	N	NA	N	CL
Bianconi et al. (2023)	Y	Y	N	PY	Y	N	N	PY	NA/PY	N	NA/Y	Y	Y	Y	Y	Y	L
Bolanis et al. (2024)	Y	PY	N	PY	Y	Y	N	PY	N/PY	N	NA/NA	NA	Y	Y	NA	Y	CL
Briggs et al. (2022)	Y	Y	Y	PY	Y	Y	N	Y	Y/NA	N	Y/NA	Y	Y	Y	N	Y	CL
Browning et al. (2022)	Y	N	N	N	Y	Y	N	N	NA/N	N	NA/NA	NA	Y	N	NA	Y	CL
Cao et al. (2023)	Y	N	N	PY	Y	Y	N	PY	NA/Y	Y	NA/Y	Y	Y	Y	Y	Y	CL
Ccami-Bernal et al. (2023)	Y	PY	N	PY	Y	Y	N	PY	NA/PY	N	NA/NA	NA	Y	Y	NA	Y	L
Chae et al. (2021)	Y	N	N	N	N	Y	N	PY	Y/PY	N	NA/NA	NA	N	N	NA	Y	CL
Chen et al. (2022)	Y	N	N	PY	Y	N	Y	PY	NA/Y	N	NA/NA	NA	Y	N	NA	Y	L
De la Fuente et al. (2021)	Y	N	N	PY	Y	Y	N	N	NA/PY	N	NA/NA	NA	Y	N	NA	Y	CL
Gao et al. (2024)	Y	PY	N	PY	Y	Y	N	PY	Y/PY	N	NA/NA	NA	N	Y	NA	Y	CL
Hsueh et al. (2022)	Y	PY	N	PY	N	N	N	N	NA/PY	N	NA/NA	NA	Y	Y	NA	Y	L
Kang et al. (2022)	Y	N	N	PY	N	N	N	N	Y/NA	N	N/NA	N	Y	Y	Y	Y	CL
Lampert et al. (2021)	Y	N	N	PY	Y	Y	N	N	NA/PY	N	NA/NA	NA	N	N	NA	Y	CL
Li et al. (2023)	Y	Y	Y	PY	Y	Y	Y	PY	NA/PY	Y	NA/Y	N	Y	Y	N	Y	L
Li and Lange (2023)	Y	N	N	N	Y	N	N	N	N/N	N	NA/NA	NA	N	N	NA	Y	CL
Masdor et al. (2023)	Y	PY	N	PY	Y	Y	N	PY	NA/PY	N	NA/NA	NA	Y	Y	NA	N	L
Patwary et al. (2024)	Y	Y	Y	PY	Y	Y	Y	PY	NA/Y	Y	NA/Y	Y	Y	Y	Y	Y	H
Piva et al. (2024)	Y	N	N	PY	Y	N	N	PY	N/N	N	NA/NA	NA	Y	N	NA	Y	CL
Qiu et al. (2022)	Y	N	Y	PY	Y	N	N	PY	Y/PY	N	Y/N	Y	Y	Y	Y	Y	CL
Rahimi-Ardabili et al. (2021)	Y	N	N	PY	Y	N	N	PY	Y/PY	N	NA/NA	NA	N	Y	NA	Y	CL
Ricciardi et al. (2022)	Y	N	N	PY	N	N	N	N	NA/PY	N	NA/NA	NA	Y	N	NA	Y	CL
Rigolon et al. (2021)	Y	N	N	PY	Y	Y	N	N	PY/PY	N	NA/NA	NA	Y	N	NA	Y	CL
Rojas-Rueda et al. (2021)	Y	N	Y	PY	Y	Y	N	N	NA/PY	N	NA/NA	NA	N	Y	NA	Y	CL
Siah et al. (2023)	Y	N	N	PY	Y	Y	N	PY	PY/N	N	Y/N	N	N	Y	N	Y	CL
Sivak et al. (2021)	Y	N	N	PY	Y	N	N	N	N/N	N	NA/NA	NA	N	N	NA	N	CL
Tang et al. (2023)	Y	Y	N	N	Y	Y	N	PY	NA/PY	N	NA/Y	N	N	Y	Y	Y	CL
Tharrey and Darmon (2021)	Y	N	N	PY	N	N	N	PY	N/PY	N	NA/NA	NA	Y	N	NA	Y	CL
Tu (2022)	Y	N	Y	PY	N	Y	N	PY	PY/NA	N	Y/NA	Y	Y	Y	Y	Y	CL
Wang et al. (2022)	Y	N	N	N	Y	Y	N	PY	NA/PY	N	NA/Y	Y	N	Y	Y	Y	CL
Wu et al. (2022)	Y	Y	N	PY	Y	Y	N	PY	NA/Y	Y	NA/Y	Y	Y	Y	Y	Y	L
Ye et al. (2022)	Y	N	Y	PY	Y	Y	N	PY	NA/PY	N	NA/Y	N	Y	N	N	N	CL
Yi et al. (2022)	Y	N	N	PY	N	N	N	N	Y/NA	N	Y/NA	N	N	N	N	Y	CL
Yuan et al. (2021)	Y	Y	N	N	Y	Y	N	PY	NA/PY	N	NA/Y	Y	N	Y	Y	Y	CL
Zagnoli et al. (2022)	Y	N	N	PY	Y	N	N	PY	NA/Y	N	NA/Y	Y	N	Y	N	Y	CL
Zare Sakhvidi, Knobel, et al. (2022a)	Y	N	N	PY	Y	N	Y	PY	NA/PY	N	NA/NA	NA	Y	Y	NA	Y	L
Zare Sakhvidi, Yang, et al. (2022b)	Y	Y	N	PY	Y	N	Y	N	NA/PY	Y	NA/Y	N	Y	Y	N	Y	CL
Zhang et al. (2021)	Y	N	N	N	N	N	N	PY	NA/PY	N	NA/NA	NA	N	Y	NA	Y	CL
Y. Zhang et al. (2024a)	Y	N	N	N	N	N	N	PY	NA/PY	N	NA/Y	Y	Y	Y	Y	Y	CL
Y. D. Zhang et al. (2024b)	Y	N	N	N	Y	Y	N	PY	N/Y	Y	NA/NA	NA	Y	Y	NA	Y	CL
X. Zhang et al. (2022c)	Y	N	N	PY	Y	Y	N	PY	PY/N	N	Y/N	Y	N	Y	Y	Y	CL
Zhao et al. (2022)	Y	Y	N	N	Y	Y	N	PY	NA/Y	Y	NA/N	Y	Y	Y	Y	Y	CL
Zhao et al. (2021)	Y	N	N	PY	Y	Y	N	PY	NA/PY	N	NA/Y	Y	Y	Y	Y	Y	CL

^a^ 1: Participant, Intervention, Comparison, Outcome (PICO) components, 2: Pre-established protocol, 3: Explanation of included studies’ design, 4: Comprehensive search strategy, 5: Study selection in duplicate, 6: Data extraction in duplicate, 7: List of excluded studies with justification, 8: Description of included studies, 9: Assessment of risk of bias (RoB) in included studies, 10: Funding sources of included studies, 11: Use of appropriate statistical methods, 12: RoB impact on synthesised results, 13: Results interpretation with RoB reference, 14: Heterogeneity explanation, 15: Publication/small study bias investigation, 16: Conflict of interest declaration.

^b^ Y = Yes; PY= Partial Yes; N = No; NA = Not applicable (no meta-analysis conducted); CL = Critically low; L = Low; H = High.

## Discussion

This umbrella review builds on prior work ( Bryer et al., 2024), highlighting growing interest in the relationship between greenspace exposure and human health ( Farkas et al., 2023). By summarising findings from 45 systematic reviews published since December 2020, we offer a comprehensive and updated overview of health outcomes linked to greenspace exposure. The results reflect both the breadth of outcomes investigated and the variability in evidence across health domains.

We found beneficial associations between greenspace exposure and a range of health outcome categories, particularly mental health ( Bonaccorsi et al., 2023; Cuijpers et al., 2023; Zare Sakhvidi et al., 2023), QoL ( Bonaccorsi et al., 2023), and cardiovascular health ( Liu et al., 2023), which were consistent with earlier studies. Compared to our previous umbrella review, which summarised studies published up to December 2020, this review observed stronger associations for cardiovascular and metabolic health, as well as general health and QoL. This review also revealed that favourable associations for birth outcomes, cardiovascular health, and mortality were more pronounced in urban areas than in less urban areas ( Browning et al., 2022). Likewise, associations between greenspace and general and cardiovascular health were more pronounced among individuals with a lower SES than those with a higher SES ( Rigolon et al., 2021). This suggests that greenspace is likely to function as a buffer against urban stressors and contribute to reducing health disparities (Bressane et al., 2024; Heo & Bell, 2023).

However, we found more inconsistent evidence for all-cause mortality, cognitive function, and cancer outcomes than in our previous review. This shift in the evidence may reflect the expansion of research in some domains. Systematic reviews examining respiratory outcomes such as asthma and AR presented mixed findings - some reporting protective associations, others showing null or conflicting associations. Similar inconsistencies were noted in prior umbrella reviews ( Yang et al., 2021; Zare Sakhvidi et al., 2023). These inconsistencies may be attributable to variability in study designs, greenspace measures, and definitions of health outcomes across original studies. Limited and inconclusive evidence was also observed for maternal health and birth outcomes ( Xie et al., 2024) and cancer outcomes ( Yang et al., 2021), restricting the strength of conclusions in these domains. Compared to our previous review, more harmful associations were reported for some health outcomes, such as communicable and vector-borne diseases, which fall under the other health outcomes category ( Rahimi-Ardabili et al., 2021; Sivak et al., 2021).

Since our previous review ( Bryer et al., 2024), we have also observed a notable increase in the number of randomised controlled trials (RCTs) evaluating greenspace interventions, particularly in relation to mental health and psychological stress. Commonly studied interventions include forest bathing, community gardening, and nature-based therapy. Although RCTs remain less prevalent in environmental health than observational designs, their emergence represents a positive shift ( Pynegar et al., 2021). Unlike observational designs, which are susceptible to residual confounding and reverse causality, RCTs allow causal inference. Therefore, the findings are more convincing for urban planning and public health interventions ( Gale et al., 2023).

Nonetheless, limitations in RCTs persist, and observational studies remain important for understanding the long-term and cumulative health effects of greenspace exposure. Many RCTs to date have small sample sizes, short durations, and lack blinding of participants and assessors, which may reduce internal validity ( Pynegar et al., 2021). In addition, the heterogeneity in intervention types, duration, and intensity makes synthesis challenging. Despite these issues, the upward trend in RCTs underscores a growth in the evidence on how greenspace interventions can promote mental health and well-being.

Modest shifts in greenspace exposure measures since our previous umbrella review were also identified. While NDVI remained the most commonly used measure, proximity to greenspace and percentage-based metrics slightly declined since our previous review. Notably, other satellite-derived indices such as the EVI and SAVI were more frequently used. Both of our umbrella reviews highlight the wide, heterogeneous range of greenspace exposure measures used which proves to be an ongoing challenge in cross-study comparability ( Freymueller et al., 2024).

Compared to our previous umbrella review, this review identified a wider breadth of health outcomes studied in relation to greenspace exposure, nearly doubling the number of health outcomes examined. In the previous review, only four systematic reviews focused on cancer outcomes, covering a limited range of cancer types. In contrast, this review identified twice as many studies on cancer, encompassing a broader spectrum of cancer types, including colorectal, breast, and oral cancers. The number of other health outcomes also grew substantially to include communicable diseases such as tuberculosis, dengue, and malaria, reflecting an increasing interest in the relationship between greenspace and infectious disease burden. This notable expansion corroborates the growing volume of research in this field ( Farkas et al., 2023).

Despite these advancements, a decline in the overall quality of systematic reviews has been observed since the onset of the COVID-19 pandemic ( Baumeister et al., 2021). Several systematic reviews included in this umbrella review lacked one or more of the following: preregistered protocols, comprehensive and reproducible search strategies, and adequate reporting of risk of bias and synthesis methods. These shortcomings reflect key departures from established best practices outlined in the ROBIS ( Whiting et al., 2016) and AMSTAR-2 guidelines ( Shea et al., 2017). This raises concerns about the reliability of recent evidence and underscores the need for high-quality systematic reviews.

The strengths of this umbrella review lie in its comprehensive approach. By conducting an extensive search across five databases, it captures a broader and more diverse range of studies compared to prior reviews ( Cuijpers et al., 2023; Xie et al., 2024; Yang et al., 2021; Zare Sakhvidi et al., 2023). The inclusion of both quantitative and qualitative evidence offers a more holistic understanding of the association between greenspace exposure and health outcomes and addresses limitations of earlier reviews that focus on quantitative data ( Xie et al., 2024). Another strength of this umbrella review is its exclusive focus on health outcomes, rather than upstream determinants such as physical activity or social cohesion. This focus ensures clearer attribution of health effects to greenspace exposure itself.

This umbrella review is also subject to several limitations that should be considered when interpreting the findings. Evidence on the association between greenspace and human health is highly heterogeneous due to differences in definitions of greenspace, exposure measures, and outcome measures across original studies and systematic reviews. This diversity limits the comparability of systematic reviews and makes it difficult to draw definitive conclusions. This umbrella review is also impacted by the generally high risk of bias and low methodological quality of included systematic reviews. Finally, while systematic reviews were included or excluded based on publication date, this was not extended to original studies. As such, this umbrella review may include systematic reviews that synthesised original studies published before December 2020, potentially leading to overlap in the original studies covered by this umbrella review and our previous umbrella review.

Mechanisms underlying the health benefits of greenspace exposure remain a critical area of inquiry. Moderators such as urbanicity and SES were explored by Browning et al. (2022) and Rigolon et al. (2021), respectively. However, mediators such as increased social cohesion ( Elliott et al., 2023; L. Zhang et al., 2022b) and decreased environmental stressors ( Chen et al., 2021; Wang & Tassinary, 2024) have been investigated in other studies. For mental health and cognitive function outcomes, interventions like community gardening and forest therapy may enhance social cohesion ( J. Zhang et al., 2022a) and promote relaxation ( Zhang et al., 2021). Social interaction facilitated by greenspaces can foster community bonds and reduce feelings of isolation, further contributing to mental well-being and cognitive function ( Nawrath et al., 2022). The aesthetic qualities of greenspaces, such as visual greenery, may also stimulate positive psychological responses ( Nawrath et al., 2022).

There are a variety of other mechanisms for the association between greenspace and human health outcomes. For cardiovascular and metabolic health, greenspaces may reduce exposure to environmental stressors such as heat and noise, while promoting independent or group physical activities ( Cardinali et al., 2023; Keith et al., 2024; Pan et al., 2024). These activities not only improve physical fitness but also regulate blood pressure, cholesterol levels, and glucose metabolism ( Keith et al., 2024). Physical activity may also act as a mediator between greenspace and mental health and cognitive function via the release of growth factors and neurotransmitters, which are important for mood, memory, and learning ( Martín-Rodríguez et al., 2024). Additionally, greenspaces may improve air quality by reducing particulate matter and other pollutants, leading to better respiratory ( Li et al., 2024) and cardiovascular ( Qiu et al., 2021) health. Investigation of causal pathways is needed to gain a thorough understanding of these mechanisms. 

The combined findings of our umbrella reviews have important implications for urban planning and public health policies. Increasing access to greenspace, particularly in urban and socioeconomically disadvantaged areas, could be a simple and cost-effective strategy to improve population health and reduce health inequalities ( Bressane et al., 2024; Rigolon et al., 2021). Integration of greenspace into healthcare interventions, such as green prescriptions has increased ( Kondo et al., 2020) and should continue to be prioritised alongside other initiatives including urban greening projects and preservation of existing greenspaces ( Bikomeye et al., 2021). Ensuring community consultation, equitable distribution, and long-term maintenance of green spaces will also be essential to maximising health benefits. This will require collaboration across sectors, including health, environment, transport, and housing, to create green spaces that are both resilient and responsive to local needs.

## Author contributions

**Brittnee Bryer:** methodology, validation, formal analysis, investigation, data curation, writing – original draft, writing – review & editing, visualisation. **Nicholas Osborne:** conceptualisation, validation, methodology, writing – review & editing, supervision. **Jialu Wang:** investigation, data curation, validation, writing – review & editing. **Rajarshi Dasgupta:** validation, writing – review & editing. **Gail Williams:** conceptualisation, methodology, writing – review & editing, supervision. **Darsy Darssan:** conceptualisation, methodology, validation, formal analysis, investigation, data curation, writing – original draft, writing – review & editing, visualisation, supervision, project administration.

## Data Availability

All data underlying the results are available as part of the article and no additional source data are required. Open Science Framework (OSF): Greenspace exposure and associated health outcomes: an updated systematic review of reviews. Supplementary Material. DOI: http://doi.org/10.17605/OSF.IO/XPZCJ This project contains the following extended data:
• Supplementary Table S1. (Literature search strategies) • Supplementary Figure S1a-h. (Risk of bias assessment by health outcome category) • Supplementary Figure S2a-h. (Overall methodological quality by health outcome category) • Supplementary Table S2. (Risk of bias assessment by domain) • Supplementary Figure S3: (Overall methodological quality) • Supplementary File S1. (Systematic reviews excluded during full-text screening) Open Science Framework (OSF): PRISMA checklist and flow chart for Greenspace exposure and associated health outcomes: an updated systematic review of reviews. DOI: 10.17605/OSF.IO/XPZCJ Data are available under the terms of the Creative Commons Attribution 4.0 International license (CC-BY 4.0). Endnote version 20 was used as a reference management tool in this study ( The EndNote Team, 2013). A free alternative to Endnote is Zotero ( Zotero, 2024). Covidence was used to streamline eligibility screening and data extraction in this study ( Covidence systematic review software, 2022). A free alternative to Covidence in Rayyan ( Ouzzani et al., 2016).
