# Supplementary material for: Greenspace exposure and associated health outcomes: an updated systematic review of reviews
Source: F1000Res. 2026 Nov 5;14:726. Originally published 2025 Jul 24. [Version 3] doi: 10.12688/f1000research.166852.3 (PMC13768706; doi:10.12688/f1000research.166852.3)
Supplement: Peer review history

# Reviewer report: Reviewer response for version 3

**DOI:** 10.5256/f1000research.213435.r538554

**Published:** 2026-11-19

**Authors:** Anna Soerensen

**Affiliations:** 1 Hasselt University, Hasselt, Belgium

I thank the authors for their work and revisions. With the implementation of the CCA's per health outcome, all of my raised points have been resolved, and I therefore happily approve the manuscript.

Are the rationale for, and objectives of, the Systematic Review clearly stated?

Yes

Is the statistical analysis and its interpretation appropriate?

Yes

If this is a Living Systematic Review, is the ‘living’ method appropriate and is the search schedule clearly defined and justified? (‘Living Systematic Review’ or a variation of this term should be included in the title.)

Not applicable

Are sufficient details of the methods and analysis provided to allow replication by others?

Partly

Are the conclusions drawn adequately supported by the results presented in the review?

Partly

Reviewer Expertise:

Environmental epidemiology

I confirm that I have read this submission and believe that I have an appropriate level of expertise to confirm that it is of an acceptable scientific standard.

# Reviewer report: Reviewer response for version 3

**DOI:** 10.5256/f1000research.213435.r538556

**Published:** 2026-11-13

**Authors:** Ben Senkler, Julius Freymueller

**Affiliations:** 1 Universitat Bielefeld Medizinische Fakultat OWL (Ringgold ID: 575404), Bielefeld, North Rhine Westfalia, Germany

We thank the authors for their constructive response and for the revisions made in version 3. All of the points we raised have been addressed.

 Our main concern is resolved: the CCA is now reported for each outcome category and each ICD-10 disease category (Table 4) alongside the overall value, with reference to Pieper et al. (2014), so that overlap is visible where it matters for interpreting the evidence. The misidentified high-quality review has been corrected to Patwary et al. (2024), the characterisation of maternal and birth outcomes is now consistent across the Abstract, Results, and Discussion, and the Abstract and Discussion now acknowledge the adverse associations reported for communicable and vector-borne diseases, giving a more balanced picture.

 The remaining minor points have likewise been addressed, and we accept the authors’ reasoning that separating intervention-based from ambient-exposure evidence would exceed the scope of an umbrella review.

 With these revisions our earlier reservations are resolved, and we are glad to approve the manuscript.

Are the rationale for, and objectives of, the Systematic Review clearly stated?

Yes

Is the statistical analysis and its interpretation appropriate?

Yes

If this is a Living Systematic Review, is the ‘living’ method appropriate and is the search schedule clearly defined and justified? (‘Living Systematic Review’ or a variation of this term should be included in the title.)

Not applicable

Are sufficient details of the methods and analysis provided to allow replication by others?

Yes

Are the conclusions drawn adequately supported by the results presented in the review?

Partly

Reviewer Expertise:

Environmental (mental) Health / environmental epidemiology

We confirm that we have read this submission and believe that we have an appropriate level of expertise to confirm that it is of an acceptable scientific standard.

# Reviewer report: Reviewer response for version 2

**DOI:** 10.5256/f1000research.198281.r485819

**Published:** 2026-06-15

**Authors:** Ben Senkler, Julius Freymueller

**Affiliations:** 1 Universitat Bielefeld Medizinische Fakultat OWL (Ringgold ID: 575404), Bielefeld, North Rhine Westfalia, Germany

Thank you for giving us the opportunity to conduct a review of the manuscript ““ *Greenspace exposure and associated health outcomes: an updated systematic review of reviews*”. We reviewed this submission for the first time at version 2 and did not assess version 1, so the comments below represent an initial overall appraisal of the current version rather than a follow-up on earlier remarks.

 This is a substantial and carefully executed umbrella review, highlighting important aspects of the interaction of greenspace exposure and health. The authors searched five databases, synthesised both quantitative and qualitative systematic reviews, and covered a broad range of health outcomes and age groups, which makes the manuscript a useful and up-to-date map of the greenspace–health literature. The dual appraisal with AMSTAR-2 and ROBIS, the ICD-10 mapping of outcomes, and the candid reporting of the predominantly low methodological quality of the evidence base are strengths of this umbrella review. In Version 2, the authors have also clearly responded earlier reviewer remarks, the expanded Tables 1 and 5, the per-outcome summary sentences, the CCA calculation, and the extended limitations are all well considered additions. We have several remaining minor comments, which we believe could further strengthen the manuscript.

1. **Corrected coverage area (CCA): the overall value is hard to interpret.** We agree with the point raised independently by Dr Anna Soerensen and Dr Mingwei Liu. A single overall CCA of 0.007 says little here, and can even be misleading. With 45 reviews spread across eight outcome categories (most covering only one or two), a low overall overlap is to be expected and does not really show that the reviews are independent. What matters is the overlap within a category, where the same primary studies can recur and make the evidence look more consistent than it is. We would therefore recommend reporting the CCA at least per outcome category (Per individual outcome would be cleaner in principle but unstable with so few reviews each, so the category level is the practical compromise.) The overall value can stay as a supplement, but with a note that a low figure is expected given the category structure.
2. **The single high-quality review appears to be misidentified.** The Results text ("Quality of evidence and risk of bias") states that only one review was rated high quality and names Piva et al. (2024). But Table 6 shows the single AMSTAR-2 "High" rating for Patwary et al. (2024) and rates Piva et al. (2024) as "Critically low". The text and Table 6 are therefore inconsistent. Please check the consistency of the text with Table 6.
3. **Inconsistent characterisation of maternal and birth outcomes across sections.** The conclusion for this category differs across the three places it is mentioned/summarized: the Abstract lists it under “ *inconsistent* associations”. The summary sentence at the end of the Results subsection states that the evidence showed *protective* associations but the Discussion describes it as *limited and inconclusive*beyond birthweight and preterm birth. We would ask the authors to harmonise the wording across the Abstract, Results, and Discussion to ensure consistence.
4. **The predominantly positive framing does not clearly reflect the documented potential harms.**The "Other health outcomes" subsection and Table 4 report several adverse or risk-increasing associations (for example elevated blood lead levels in children and a higher prevalence of West Nile virus-carrying mosquitoes around green vacant lots, and an increased incidence of malaria, dengue, and tuberculosis in one review, alongside dermatophytosis and toxocariasis). These are not reflected in the Abstract or in the overall framing of the Discussion, which present greenspace as uniformly beneficial. Inconsistent results are transparently addressed in the discussion, but we would encourage the authors to consider acknowledging the documented potential harms, so that the manuscript presents an even more balanced picture.

**Small adaptations**

1. **Table 3 denominator.** The column header reads "% out of 44 reviews," but the percentages are calculated on 45 reviews (e.g., NDVI: 27/45 = 60.0%; 27/44 would be 61.4%). The header should be corrected to 45.
2. **Causal/evaluative language.** "Protective," and "favourable" are used throughout the Abstract, Results, and Discussion to describe associations drawn from an evidence base that is largely cross-sectional (45.7% of original studies: Table 2) for example, and several subsections describe "protective" effects. "Protective" in particular implies a causal, risk-reducing relationship that cross-sectional data cannot establish. We would invite the authors to consider softening some of this wording toward more neutral associational language, and to decide for themselves how far to adjust it.
3. **Cortisol terminology and the CI.** In "Other health outcomes," the salivary cortisol result from Qiu et al. (2022) is described as "serum cortisol concentration (SCC)" with a 95% CI of (-1.10, -0.04), whereas Table 5 labels the same result "salivary cortisol concentration" with a CI of (-0.10, -0.04); the abbreviation list also defines SCC as serum cortisol. As far as we see, the underlying study measured salivary cortisol, so "serum" appears to be an error, and the lower CI bound in the text (-1.10) is inconsistent with Table 5 (-0.10). Please check.
4. **Cognitive function.** The Abstract groups cognition within "mental health" (rated beneficial), whereas the Discussion lists "cognitive function" among the outcomes for which the evidence was more inconsistent. The signaling on cognition could be made consistent between the two sections.
5. **Maternal/birth review count text versus Table 4.** The Results state that five reviews investigated maternal health and birth outcomes and name Rojas-Rueda et al. (2021) among them, but the maternal/birth section of Table 4 lists only four reviews (Rojas-Rueda's outcome, infant mortality, appears under the mortality category). Please check if you would like to adjust it.
6. **Distinction between intervention and ambient-exposure evidence.** The summary verdicts for some categories (mental health, for example) draw on two quite different kinds of evidence: observational studies of residential or surrounding greenspace (e.g., NDVI in cross-sectional studies) and intervention studies such as forest bathing or gardening (often randomised). These differ both in the type of exposure and in how strongly they support causal claims, so a single combined verdict can blur that distinction. The authors may want to consider whether and how to separate these two strands or mention this conflation.
7. **Reporting guideline.** The Methods cite PRISMA 2009 (Moher et al., 2009). For an overview of reviews, the dedicated PRIOR statement (Gates et al., 2022) and/or the updated PRISMA 2020 statement (Page et al., 2021) are the current reporting standards, and adherence to one of these would be preferable.
8. **Citation of ROBIS.** In the Methods, ROBIS is cited as "(Shea et al., 2017)"; ROBIS is Whiting et al. (2016) (as correctly cited later in the Discussion and in the reference list) while Shea et al. (2017) is the AMSTAR-2 reference. Please adjust the citation accordingly.
9. **Addition of physical activity as an important mediating factor.** The Discussion presents a comprehensive overview of moderators and mediators regarding the impact of greenspace exposure on health outcomes. Physical activity is mentioned here only as a relevant mechanism for cardiovascular and metabolic health, although it is stated in the Results as a mediator for mental health. Please consider adding physical activity to the discussion of potential mediators.

Are the rationale for, and objectives of, the Systematic Review clearly stated?

Yes

Is the statistical analysis and its interpretation appropriate?

Yes

If this is a Living Systematic Review, is the ‘living’ method appropriate and is the search schedule clearly defined and justified? (‘Living Systematic Review’ or a variation of this term should be included in the title.)

Not applicable

Are sufficient details of the methods and analysis provided to allow replication by others?

Yes

Are the conclusions drawn adequately supported by the results presented in the review?

Partly

Reviewer Expertise:

Environmental (mental) Health / environmental epidemiology

We confirm that we have read this submission and believe that we have an appropriate level of expertise to confirm that it is of an acceptable scientific standard, however we have significant reservations, as outlined above.

# Response

**Published:** 2026-10-23

**Authors:** Darsy Darssan

**Affiliations:** The University of Queensland, Australia

We thank the reviewers for their thorough and insightful review of our manuscript. We greatly appreciate their positive assessment of the review's methodological approach and reporting, as well as their detailed suggestions, which have helped us further improve the manuscript.

**Thank you for giving us the opportunity to conduct a review of the manuscript ““ *Greenspace exposure and associated health outcomes: an updated systematic review of reviews*”. We reviewed this submission for the first time at version 2 and did not assess version 1, so the comments below represent an initial overall appraisal of the current version rather than a follow-up on earlier remarks.**

**This is a substantial and carefully executed umbrella review, highlighting important aspects of the interaction of greenspace exposure and health. The authors searched five databases, synthesised both quantitative and qualitative systematic reviews, and covered a broad range of health outcomes and age groups, which makes the manuscript a useful and up-to-date map of the greenspace–health literature. The dual appraisal with AMSTAR-2 and ROBIS, the ICD-10 mapping of outcomes, and the candid reporting of the predominantly low methodological quality of the evidence base are strengths of this umbrella review. In Version 2, the authors have also clearly responded earlier reviewer remarks, the expanded Tables 1 and 5, the per-outcome summary sentences, the CCA calculation, and the extended limitations are all well considered additions. We have several remaining minor comments, which we believe could further strengthen the manuscript.**

**1. Corrected coverage area (CCA): the overall value is hard to interpret. We agree with the point raised independently by Dr Anna Soerensen and Dr Mingwei Liu. A single overall CCA of 0.007 says little here, and can even be misleading. With 45 reviews spread across eight outcome categories (most covering only one or two), a low overall overlap is to be expected and does not really show that the reviews are independent. What matters is the overlap within a category, where the same primary studies can recur and make the evidence look more consistent than it is. We would therefore recommend reporting the CCA at least per outcome category (Per individual outcome would be cleaner in principle but unstable with so few reviews each, so the category level is the practical compromise.) The overall value can stay as a supplement, but with a note that a low figure is expected given the category structure.**

 We calculated the CCA separately for each health outcome category and incorporated these findings into the results section. We also calculated CCA values for each ICD disease category and included these results in Table 4.

**2. The single high-quality review appears to be misidentified. The Results text ("Quality of evidence and risk of bias") states that only one review was rated high quality and names Piva et al. (2024). But Table 6 shows the single AMSTAR-2 "High" rating for Patwary et al. (2024) and rates Piva et al. (2024) as "Critically low". The text and Table 6 are therefore inconsistent. Please check the consistency of the text with Table 6.**

 We reviewed the AMSTAR-2 ratings and confirmed that the single review rated as high quality was Patwary et al. (2024). The text has been corrected to align with Table 6.

**3. Inconsistent characterisation of maternal and birth outcomes across sections. The conclusion for this category differs across the three places it is mentioned/summarized: the Abstract lists it under “ *inconsistent* associations”. The summary sentence at the end of the Results subsection states that the evidence showed *protective* associations but the Discussion describes it as *limited and inconclusive*beyond birthweight and preterm birth. We would ask the authors to harmonise the wording across the Abstract, Results, and Discussion to ensure consistence.**

 We have reviewed the wording in the abstract, results, and discussion, and revised the text to ensure a consistent interpretation of the evidence for maternal health and birth outcomes across all sections of the manuscript.

**4. The predominantly positive framing does not clearly reflect the documented potential harms. The "Other health outcomes" subsection and Table 4 report several adverse or risk-increasing associations (for example elevated blood lead levels in children and a higher prevalence of West Nile virus-carrying mosquitoes around green vacant lots, and an increased incidence of malaria, dengue, and tuberculosis in one review, alongside dermatophytosis and toxocariasis). These are not reflected in the Abstract or in the overall framing of the Discussion, which present greenspace as uniformly beneficial. Inconsistent results are transparently addressed in the discussion, but we would encourage the authors to consider acknowledging the documented potential harms, so that the manuscript presents an even more balanced picture.**

 We have revised the abstract and the discussion (third paragraph) to better reflect both the inconsistent and potentially adverse associations identified for the other health outcomes category.

**Small adaptations**

**1. Table 3 denominator. The column header reads "% out of 44 reviews," but the percentages are calculated on 45 reviews (e.g., NDVI: 27/45 = 60.0%; 27/44 would be 61.4%). The header should be corrected to 45.**

 The denominator in the Table 3 column header has been corrected from 44 to 45 reviews.

**2. Causal/evaluative language. "Protective," and "favourable" are used throughout the Abstract, Results, and Discussion to describe associations drawn from an evidence base that is largely cross-sectional (45.7% of original studies: Table 2) for example, and several subsections describe "protective" effects. "Protective" in particular implies a causal, risk-reducing relationship that cross-sectional data cannot establish. We would invite the authors to consider softening some of this wording toward more neutral associational language, and to decide for themselves how far to adjust it.**

 We agree that causal language should be used carefully. The terms "protective association" and "favourable association" are commonly used in epidemiological literature to describe the direction of an observed association and do not necessarily imply causation. We have reviewed the manuscript to ensure that findings are consistently presented as associations rather than definitive causal effects where appropriate.

**3. Cortisol terminology and the CI. In "Other health outcomes," the salivary cortisol result from Qiu et al. (2022) is described as "serum cortisol concentration (SCC)" with a 95% CI of (-1.10, -0.04), whereas Table 5 labels the same result "salivary cortisol concentration" with a CI of (-0.10, -0.04); the abbreviation list also defines SCC as serum cortisol. As far as we see, the underlying study measured salivary cortisol, so "serum" appears to be an error, and the lower CI bound in the text (-1.10) is inconsistent with Table 5 (-0.10). Please check.**

 We have corrected the terminology and revised the confidence interval value to ensure consistency between the text and tables.

**4. Cognitive function. The Abstract groups cognition within "mental health" (rated beneficial), whereas the Discussion lists "cognitive function" among the outcomes for which the evidence was more inconsistent. The signaling on cognition could be made consistent between the two sections.**

 We have reviewed and revised the abstract to ensure that the interpretation of findings relating to cognitive function is presented consistently.

**5. Maternal/birth review count text versus Table 4. The Results state that five reviews investigated maternal health and birth outcomes and name Rojas-Rueda et al. (2021) among them, but the maternal/birth section of Table 4 lists only four reviews (Rojas-Rueda's outcome, infant mortality, appears under the mortality category). Please check if you would like to adjust it.**

 We reviewed the classification of reviews by outcome category and revised the text to ensure consistency between the results section and Table 4.

**6. Distinction between intervention and ambient-exposure evidence. The summary verdicts for some categories (mental health, for example) draw on two quite different kinds of evidence: observational studies of residential or surrounding greenspace (e.g., NDVI in cross-sectional studies) and intervention studies such as forest bathing or gardening (often randomised). These differ both in the type of exposure and in how strongly they support causal claims, so a single combined verdict can blur that distinction. The authors may want to consider whether and how to separate these two strands or mention this conflation.**

 We agree that intervention-based studies and studies of ambient or residential greenspace exposure represent distinct types of evidence and may differ in their implications for causal inference. However, the aim of this umbrella review was to synthesise and summarise findings as reported by the included systematic reviews and meta-analyses, rather than to re-classify or re-synthesise the underlying primary studies according to exposure type. As many of the included reviews synthesised both intervention and observational evidence, separating these strands would have required substantial re-analysis beyond the scope of an umbrella review.

**7. Reporting guideline. The Methods cite PRISMA 2009 (Moher et al., 2009). For an overview of reviews, the dedicated PRIOR statement (Gates et al., 2022) and/or the updated PRISMA 2020 statement (Page et al., 2021) are the current reporting standards, and adherence to one of these would be preferable.**

 PRISMA 2020 was used for this review, and the reference has been updated accordingly.

**8. Citation of ROBIS. In the Methods, ROBIS is cited as "(Shea et al., 2017)"; ROBIS is Whiting et al. (2016) (as correctly cited later in the Discussion and in the reference list) while Shea et al. (2017) is the AMSTAR-2 reference. Please adjust the citation accordingly.**

 The ROBIS reference has been corrected from Shea et al. (2017) to Whiting et al. (2016).

**9. Addition of physical activity as an important mediating factor. The Discussion presents a comprehensive overview of moderators and mediators regarding the impact of greenspace exposure on health outcomes. Physical activity is mentioned here only as a relevant mechanism for cardiovascular and metabolic health, although it is stated in the Results as a mediator for mental health. Please consider adding physical activity to the discussion of potential mediators.**

 We have expanded the discussion (twelfth paragraph) to acknowledge the role of physical activity as a potential mechanism linking greenspace exposure and mental health and cognitive function.

# Reviewer report: Reviewer response for version 2

**DOI:** 10.5256/f1000research.198281.r485825

**Published:** 2026-06-15

**Authors:** Emma J Hudgins

**Affiliations:** 1 The University of Melbourne, Parkville, Australia

This article by Bryer and colleagues conducts a review of reviews on the evidence for associations between a range of health outcomes and exposure to green spaces. This umbrella review is an update to an existing umbrella review and follows the same methods. It includes qualitative findings as well as a summary of all meta-analytic results available on these associations. The authors found that greenspace exposure had well supported positive links to mental health, general health and quality of life, and cardiovascular and metabolic health. Greenspace had more variable associations with cancer, respiratory health and allergies, and maternal health and birth outcomes. In spite of these strong linkages described across included reviews, the authors caution that methodological rigour is low across included studies and is in decline.

 This article is strong in its methodological reproducibility and adherence to FAIR principles- especially given the pre-registration of its protocol and following the revisions requested by the first round of reviewers e.g. including the search string in this article rather than only pointing a previous article. I also recognize the review’s novelty in combining synthesis from both conventional medical studies and more interdisciplinary analyses.  Its message around continuing to strive for preregistration and other important research practices that have declined in recent years is an important one and the authors here practice what they preach. The slight weakness of the paper are the need for a bit more methodological information and wording that seems to suggest a hierarchy of disciplinary knowledge surrounding the issue being studied.

 More specifically, beyond the additional concerns raised by the other reviewers, I have two of my own **minor concerns**

 1) the V1 reviewer 1 comment regarding whether you conducted forward and backward review of references contained within reviews was not addressed by the revised wording around search terms in the supplementary table. Could you add additional clarification around this?

 2) The discussion section sets up RCTs as the ideal study design, potentially unfairly, given the challenges in holistically capturing green space exposure in a feasible RCT. I think lines of evidence spanning a range of study designs adds a degree of holism to the umbrella review and could be framed more positively.

Are the rationale for, and objectives of, the Systematic Review clearly stated?

Yes

Is the statistical analysis and its interpretation appropriate?

Partly

If this is a Living Systematic Review, is the ‘living’ method appropriate and is the search schedule clearly defined and justified? (‘Living Systematic Review’ or a variation of this term should be included in the title.)

Not applicable

Are sufficient details of the methods and analysis provided to allow replication by others?

Yes

Are the conclusions drawn adequately supported by the results presented in the review?

Yes

Reviewer Expertise:

Urban ecology, biodiversity conservation

I confirm that I have read this submission and believe that I have an appropriate level of expertise to confirm that it is of an acceptable scientific standard, however I have significant reservations, as outlined above.

# Response

**Published:** 2026-10-23

**Authors:** Darsy Darssan

**Affiliations:** The University of Queensland, Australia

We thank the reviewer for their thoughtful and constructive comments, as well as their positive assessment of the strengths of this umbrella review. We appreciate the recognition of our efforts to enhance methodological transparency and reproducibility and have addressed the specific comments below.

**This article by Bryer and colleagues conducts a review of reviews on the evidence for associations between a range of health outcomes and exposure to green spaces. This umbrella review is an update to an existing umbrella review and follows the same methods. It includes qualitative findings as well as a summary of all meta-analytic results available on these associations. The authors found that greenspace exposure had well supported positive links to mental health, general health and quality of life, and cardiovascular and metabolic health. Greenspace had more variable associations with cancer, respiratory health and allergies, and maternal health and birth outcomes. In spite of these strong linkages described across included reviews, the authors caution that methodological rigour is low across included studies and is in decline.**

**This article is strong in its methodological reproducibility and adherence to FAIR principles- especially given the pre-registration of its protocol and following the revisions requested by the first round of reviewers e.g. including the search string in this article rather than only pointing a previous article. I also recognize the review’s novelty in combining synthesis from both conventional medical studies and more interdisciplinary analyses.  Its message around continuing to strive for preregistration and other important research practices that have declined in recent years is an important one and the authors here practice what they preach. The slight weakness of the paper are the need for a bit more methodological information and wording that seems to suggest a hierarchy of disciplinary knowledge surrounding the issue being studied.**

**More specifically, beyond the additional concerns raised by the other reviewers, I have two of my own minor concerns**

**1) the V1 reviewer 1 comment regarding whether you conducted forward and backward review of references contained within reviews was not addressed by the revised wording around search terms in the supplementary table. Could you add additional clarification around this?**

 Forward and background reference searching was conducted to align with the methods of our previous review, but no additional studies were identified.

**2) The discussion section sets up RCTs as the ideal study design, potentially unfairly, given the challenges in holistically capturing green space exposure in a feasible RCT. I think lines of evidence spanning a range of study designs adds a degree of holism to the umbrella review and could be framed more positively.**

 We have revised the discussion (fifth paragraph of the discussion section) to acknowledge the value of observational studies in evaluating the long-term and cumulative health effects of greenspace exposure.

# Reviewer report: Reviewer response for version 2

**DOI:** 10.5256/f1000research.198281.r476283

**Published:** 2026-05-08

**Authors:** Anna Soerensen

**Affiliations:** 1 Hasselt University, Hasselt, Belgium

I thank the authors for implementing my previous suggestions. The revisions have enhanced the clarity of the manuscript, making it straightforward to follow throughout. However, I still have one comment: Could the authors clarify the rationale for calculating the overall CCA rather than per category? Low overall overlap is somewhat expected, as most review studies only span one or two categories out of the 8 included. This might not accurately reflect the degree of overlap within each category, where it's most relevant for interpreting the evidence.

Are the rationale for, and objectives of, the Systematic Review clearly stated?

Yes

Is the statistical analysis and its interpretation appropriate?

Yes

If this is a Living Systematic Review, is the ‘living’ method appropriate and is the search schedule clearly defined and justified? (‘Living Systematic Review’ or a variation of this term should be included in the title.)

Not applicable

Are sufficient details of the methods and analysis provided to allow replication by others?

Partly

Are the conclusions drawn adequately supported by the results presented in the review?

Partly

Reviewer Expertise:

Environmental epidemiology

I confirm that I have read this submission and believe that I have an appropriate level of expertise to confirm that it is of an acceptable scientific standard, however I have significant reservations, as outlined above.

# Response

**Published:** 2026-10-23

**Authors:** Darsy Darssan

**Affiliations:** The University of Queensland, Australia

We thank the reviewer for their ongoing engagement with our manuscript and for their valuable feedback.

**I thank the authors for implementing my previous suggestions. The revisions have enhanced the clarity of the manuscript, making it straightforward to follow throughout. However, I still have one comment: Could the authors clarify the rationale for calculating the overall CCA rather than per category? Low overall overlap is somewhat expected, as most review studies only span one or two categories out of the 8 included. This might not accurately reflect the degree of overlap within each category, where it's most relevant for interpreting the evidence.**

 We have calculated the CCA separately for each health outcome category and incorporated these findings into the results section. We also calculated CCA values for each ICD disease category and included these results in Table 4.

# Reviewer report: Reviewer response for version 2

**DOI:** 10.5256/f1000research.198281.r476281

**Published:** 2026-04-23

**Authors:** Mingwei Liu

**Affiliations:** 1 Huazhong University of Science and Technology, Wuhan, China

I appreciate that the authors have nicely addressed my previous comments. I still have one comment regarding the quality and bias assessment. 

 This is feedback on points 3 and 5 of my previous comments.  

 To point 3: The CCA should be calculated for each outcome. Because the results were synthesized for each outcome. The CCA shall demonstrate the degree of overlap of primary studies that was synthesized. And if there is a large overlap for an outcome, authors can choose the input studies. 

 To point 5: The results were interpreted for each health outcome. Therefore, authors need to know the quality and bias of each result by outcome. The quality and bias should be analyzed for each outcome, so that it is more informative for future studies.

Are the rationale for, and objectives of, the Systematic Review clearly stated?

Partly

Is the statistical analysis and its interpretation appropriate?

Yes

If this is a Living Systematic Review, is the ‘living’ method appropriate and is the search schedule clearly defined and justified? (‘Living Systematic Review’ or a variation of this term should be included in the title.)

Not applicable

Are sufficient details of the methods and analysis provided to allow replication by others?

Partly

Are the conclusions drawn adequately supported by the results presented in the review?

Yes

Reviewer Expertise:

Environmental epidemiology

I confirm that I have read this submission and believe that I have an appropriate level of expertise to confirm that it is of an acceptable scientific standard, however I have significant reservations, as outlined above.

# Response

**Published:** 2026-10-23

**Authors:** Darsy Darssan

**Affiliations:** The University of Queensland, Australia

We thank the reviewer for their continued engagement with our manuscript and for their constructive comments. We appreciate the thoughtful suggestions regarding overlap, methodological quality, and risk of bias assessments, which have helped strengthen the transparency and interpretation of our findings.

**I appreciate that the authors have nicely addressed my previous comments. I still have one comment regarding the quality and bias assessment.**

**This is feedback on points 3 and 5 of my previous comments.**

**To point 3: The CCA should be calculated for each outcome. Because the results were synthesized for each outcome. The CCA shall demonstrate the degree of overlap of primary studies that was synthesized. And if there is a large overlap for an outcome, authors can choose the input studies.**

 We have calculated the CCA separately for each health outcome category and incorporated these findings into the results section. We also calculated CCA values for each ICD disease category and included these results in Table 4.

**To point 5: The results were interpreted for each health outcome. Therefore, authors need to know the quality and bias of each result by outcome. The quality and bias should be analyzed for each outcome, so that it is more informative for future studies.**

 We have summarised the methodological quality and risk of bias of the included reviews by health outcome category and added these results to the Supplementary Materials.

# Reviewer report: Reviewer response for version 1

**DOI:** 10.5256/f1000research.183900.r444035

**Published:** 2026-01-13

**Authors:** Anna Soerensen

**Affiliations:** 1 Hasselt University, Hasselt, Belgium

Thank you for giving me the opportunity to review the manuscript entitled *“Greenspace exposure and associated health outcomes: an updated systematic review of reviews”.* This umbrella review provides an overview of the latest reviews within the field of green space exposure and health outcomes. Overall, the manuscript succeeds in providing this overview and clearly reflects the substantial effort invested in its preparation. It also highlights the important observation that the vast majority of included systematic reviews and meta-analyses are of low quality. Additionally, the structure and writing are clear and well organized, making the manuscript a pleasure to read. However, I do have some comments.

**Major comments:**

1. The search strategy is not entirely clear. Referring to your previous review article gives the impression that the full search strategy can be found there. For instance, in your previous review you state: *“To ensure that relevant systematic reviews were retrieved, we also conducted forward and backward reference searching on screened reviews, as well as manual searching of reference lists of relevant reviews”, *which makes it unclear whether this was also conducted for the current manuscript. I would also recommend explicitly stating that the search terms and strategies can be found in Supplementary Table S1, and perhaps providing a few examples of the search terms used.
2. This is a well-conducted umbrella review, but it would be markedly strengthened by an assessment of study overlap. If no assessment of study overlap is conducted, I would suggest including a statement in the limitations section and/or providing a rationale for why this was not considered relevant.
3. Including age groups and geographical location in Table 1 would further strengthen the manuscript, particularly as coverage across all age groups is one of the rationales for conducting this review.

**Minor comments:**

1. The abstract is well written, to the point, and correctly summarizes the findings of the manuscript. My only suggestion is to add *“green space”* and " *greenness*" as keywords, if possible.
2. In the methods section, I would appreciate clarification regarding *“iv) reported health outcome(s) directly attributable to greenspace exposure”*. 
3. The results section has a logical flow and is easy to follow. However, I am missing a definition of *“overall greenspace exposure”* and *“forest-based interventions”*. It is not clear which types of studies are included under these terms.
4. On page 4, the sentence *“with mediating factors such as air quality, perceived stress, and physical activity identified (Zhang et al., 2021)”* is included, but Zhang et al. (2021) is not mentioned in the preceding sentence, making it unclear which type of green space exposure they assessed.
5. I would consider adding one or two sentences at the end of each outcome subsection summarizing the overall findings. Almost all outcome subsections include at least one review article that does not align with the findings of the other reviews, which makes it difficult to determine whether there is an overall effect. While you do summarize the main findings and conclusions in the discussion, I believe it would be helpful to include such summary statements throughout the results section, for example at the end of the *“Cardiovascular and metabolic outcomes”* subsection.
6. The findings suggesting that associations may be moderated by SES, urbanicity, or related contextual factors are interesting and add nuance to the results. However, as this aspect was not described in the methods section, its inclusion in the results came somewhat unexpectedly. Clarifying this in the methods and/or presenting these findings in a dedicated subsection of the results would improve  readability
7. On page 34, you state: *“Similarly, a 0.1-unit increment in NDVI was associated with a reduced risk of asthma incidence (Tang et al., 2023)”*, which gives the impression that an odds ratio or another effect estimate is missing.
8. On page 35, *“Gardening interventions were associated with better physical health”* was stated. Could you clarify how physical health was defined?

Are the rationale for, and objectives of, the Systematic Review clearly stated?

Yes

Is the statistical analysis and its interpretation appropriate?

Yes

If this is a Living Systematic Review, is the ‘living’ method appropriate and is the search schedule clearly defined and justified? (‘Living Systematic Review’ or a variation of this term should be included in the title.)

Not applicable

Are sufficient details of the methods and analysis provided to allow replication by others?

Partly

Are the conclusions drawn adequately supported by the results presented in the review?

Partly

Reviewer Expertise:

Environmental epidemiology

I confirm that I have read this submission and believe that I have an appropriate level of expertise to confirm that it is of an acceptable scientific standard, however I have significant reservations, as outlined above.

# Response

**Published:** 2026-03-31

**Authors:** Darsy Darssan

**Affiliations:** The University of Queensland, Australia

We are thankful for your thorough and valuable comments on our manuscript. We have carefully reviewed and integrated your recommendations to the extent possible.

**Thank you for giving me the opportunity to review the manuscript entitled *“Greenspace exposure and associated health outcomes: an updated systematic review of reviews”. *This umbrella review provides an overview of the latest reviews within the field of green space exposure and health outcomes. Overall, the manuscript succeeds in providing this overview and clearly reflects the substantial effort invested in its preparation. It also highlights the important observation that the vast majority of included systematic reviews and meta-analyses are of low quality. Additionally, the structure and writing are clear and well organized, making the manuscript a pleasure to read. However, I do have some comments.**

**Major comments:**

**1. The search strategy is not entirely clear. Referring to your previous review article gives the impression that the full search strategy can be found there. For instance, in your previous review you state: *“To ensure that relevant systematic reviews were retrieved, we also conducted forward and backward reference searching on screened reviews, as well as manual searching of reference lists of relevant reviews”, *which makes it unclear whether this was also conducted for the current manuscript. I would also recommend explicitly stating that the search terms and strategies can be found in Supplementary Table S1, and perhaps providing a few examples of the search terms used.**

 We have updated the methods section to ensure that the location of the search strategy is stated more explicitly. 

**2. This is a well-conducted umbrella review, but it would be markedly strengthened by an assessment of study overlap. If no assessment of study overlap is conducted, I would suggest including a statement in the limitations section and/or providing a rationale for why this was not considered relevant.**

 We have calculated the CCA value and have added this and its interpretation to the article.

**3. Including age groups and geographical location in Table 1 would further strengthen the manuscript, particularly as coverage across all age groups is one of the rationales for conducting this review.**

 We have added the age range studies in each systematic review to the fifth column of Table 1. We have also added the location(s) covered by each systematic review to the second column of Table 1. Due to the large number and wide variety of countries covered by systematic reviews, we chose to report study location by continent instead.

****

**Minor comments:**

**1. The abstract is well written, to the point, and correctly summarizes the findings of the manuscript. My only suggestion is to add *“green space” *and " *greenness*" as keywords, if possible.**

 Thank you for this suggestion.

**2. In the methods section, I would appreciate clarification regarding “iv) reported health outcome(s) directly attributable to greenspace exposure”.**

 We have clarified this criterion in the methods section by noting that eligible reviews needed to examine health outcomes specifically attributable to greenspace exposure, independent of blue space.

**3. The results section has a logical flow and is easy to follow. However, I am missing a definition of “overall greenspace exposure” and “forest-based interventions”. It is not clear which types of studies are included under these terms.**

 We have added an explanation of what these terms encompass.

**4. On page 4, the sentence “with mediating factors such as air quality, perceived stress, and physical activity identified (Zhang et al., 2021)” is included, but Zhang et al. (2021) is not mentioned in the preceding sentence, making it unclear which type of green space exposure they assessed.**

 We have revised this section to improve clarity.

**5. I would consider adding one or two sentences at the end of each outcome subsection summarizing the overall findings. Almost all outcome subsections include at least one review article that does not align with the findings of the other reviews, which makes it difficult to determine whether there is an overall effect. While you do summarize the main findings and conclusions in the discussion, I believe it would be helpful to include such summary statements throughout the results section, for example at the end of the “Cardiovascular and metabolic outcomes” subsection.**

 We have added a sentence at the end of each health outcome section to summarise the results.

**6. The findings suggesting that associations may be moderated by SES, urbanicity, or related contextual factors are interesting and add nuance to the results. However, as this aspect was not described in the methods section, its inclusion in the results came somewhat unexpectedly. Clarifying this in the methods and/or presenting these findings in a dedicated subsection of the results would improve readability**

 The findings related to SES and urbanicity as moderators of the association between greenspace and human health outcomes were not part of the predefined objectives of the umbrella review. For this reason, we have not added them to the methods section or expanded their presentation in the results section. We have kept them only as brief contextual notes to avoid over‑interpreting the evidence.

**7. On page 34, you state: “Similarly, a 0.1-unit increment in NDVI was associated with a reduced risk of asthma incidence (Tang et al., 2023)”, which gives the impression that an odds ratio or another effect estimate is missing.**

 We have added the effect estimate for this association.

**8. On page 35, “Gardening interventions were associated with better physical health” was stated. Could you clarify how physical health was defined?**

 Physical health was generally self-reported and encompassed perceived general health, acute health complaints, physical constraints, and chronic illnesses.

# Reviewer report: Reviewer response for version 1

**DOI:** 10.5256/f1000research.183900.r433260

**Published:** 2025-12-27

**Authors:** Mingwei Liu

**Affiliations:** 1 Huazhong University of Science and Technology, Wuhan, China

The current umbrella review updated the current evidence of the association between greenspace exposure and various health outcomes. The review is conducted in a systematic approach and has a comprehensive coverage of evidence. However, some measures like corrected covered area (CCA), robustness of evidence, and time scale can be added when synthesizing the evidence. The implications should be more specific. 

 Major

 1. Are there reviews of a topic (health outcome) that were identified only in the previous umbrella review you conducted, but not in this updated review? 

 2. This umbrella review builds upon your previous review. It is good to specify that the previous review included publications between January 2010 and December 2020 at the beginning. 

 3. Have you considered the overlap of primary studies in the included systematic review? It is usually assessed by a measure of CCA. If some reviews had a high level of overlap of primary studies, you may consider only including one of them. 

 4. The study should provide the specific search strategy (the combination of search terms) in a supplementary file or clearly refer to where to find it. 

 5. The methodological quality, risk of bias, and robustness of each evidence are not reported in the Results text. It is clearer to label them alongside the evidence. It is the same for table 1 and table 5. Table 6 can be put into the supplementary file. The overall quality, risk of bias, and robustness of each evidence should be made clear in the corresponding text and tables. 

 6. What about the robustness of each quantitative evidence? The robustness comprehensively reflects the magnitude of the p-value, the sample size, the heterogeneity, and the risk of bias. There is a simple rule for assessing robustness suggested before. Please refer to the literature below. 

     1. Aromataris E, Fernandez R, Godfrey CM, Holly C, Khalil H, Tungpunkom P. Summarizing systematic reviews: methodological development, conduct and reporting of an umbrella review approach. Int J Evid Based Healthc 2015;13:132–140.

     2. Aromataris E, Fernandez RS, Godfrey CHolly C, Khalil H, Tungpunkom P. Methodology for JBI umbrella reviews 2014;1.

     3. Cleophas TJ, Zwinderman AH. Meta-meta-analysis. In: Modern meta-analysis: review and update of methodologies. Cham: Springer International Publishing; 2017, p135–143.

     4. Fusar-Poli P, Radua J. Ten simple rules for conducting umbrella reviews. Evid Based Ment Health 2018;21:95–100.

 7. Even though the included systematic reviews are different between the current umbrella review and the previous umbrella review, the included original studies (evidence) of the current umbrella review should cover most of the original studies in the previous umbrella review. Because the included systematic review did not set the criteria to include only original studies between December 2020 and June 2024, rather, they should have included earlier original studies. How do you interpret this? The readers should be aware of it when interpreting results. 

 8. The current review included a wide range of diseases and various study designs. The effective exposure time of green space may vary among diseases. For example, the time scale of the association between green space and CVD is usually years, whereas the time scale of the association between green space and mental health can be days. This should be reported in tables and results. And it could be an interesting discussion point. 

 9. What is the limitation of the current study? 

 10. The authors should give more implications for policy and future studies regarding health domains, respectively. 

 Minor

 1. In the first paragraph of the Introduction, you said "However, limitations in the methodology of these umbrella reviews have resulted in some gaps in the literature." But not all of the listed reviews are umbrella review. You can consider to rephrase it to "these umbrella or systematic reviews". 

 2. In the second paragraph of the Introduction, you first said "these reviews often present their findings narratively", then you gave an example review that "focused solely on quantitative results". It is not clear what you suggest from here? Should study include both quantitative and qualitative analyses? Or maybe you want to demonstrate that previous reviews did not include both quantitative and qualitative results. But you should give more example reviews. 

 3. Some texts are not well cited. For example, in the Discussion section, "Systematic reviews examining respiratory outcomes such as asthma and AR presented mixed findings - some reporting protective effects, others showing null or conflicting associations". Please examine thoroughly to make sure the texts have citations where it is necessary.

Are the rationale for, and objectives of, the Systematic Review clearly stated?

Partly

Is the statistical analysis and its interpretation appropriate?

Yes

If this is a Living Systematic Review, is the ‘living’ method appropriate and is the search schedule clearly defined and justified? (‘Living Systematic Review’ or a variation of this term should be included in the title.)

Not applicable

Are sufficient details of the methods and analysis provided to allow replication by others?

Partly

Are the conclusions drawn adequately supported by the results presented in the review?

Yes

Reviewer Expertise:

Environmental epidemiology

I confirm that I have read this submission and believe that I have an appropriate level of expertise to confirm that it is of an acceptable scientific standard, however I have significant reservations, as outlined above.

# Response

**Published:** 2026-03-31

**Authors:** Darsy Darssan

**Affiliations:** The University of Queensland, Australia

We sincerely appreciate the thorough and insightful feedback on the manuscript. We have carefully considered and incorporated your suggestions wherever feasible.

**The current umbrella review updated the current evidence of the association between greenspace exposure and various health outcomes. The review is conducted in a systematic approach and has a comprehensive coverage of evidence. However, some measures like corrected covered area (CCA), robustness of evidence, and time scale can be added when synthesizing the evidence. The implications should be more specific.**

**Major**

**1. Are there reviews of a topic (health outcome) that were identified only in the previous umbrella review you conducted, but not in this updated review?**

 While the major health outcome categories remained consistent with those in our previous umbrella review, some individual outcomes did not appear in this umbrella review. These include gestational age, pregnancy complications, head circumference, atherosclerosis, hypotension, oxidative stress, and inflammation markers. However, it is important to note that these outcomes may have been investigated under larger categories, such as overall cardiovascular health and physiological outcomes, by systematic reviews.

**2. This umbrella review builds upon your previous review. It is good to specify that the previous review included publications between January 2010 and December 2020 at the beginning.**

 We have added the date range of our previous umbrella review to the methods section.

**3. Have you considered the overlap of primary studies in the included systematic review? It is usually assessed by a measure of CCA. If some reviews had a high level of overlap of primary studies, you may consider only including one of them.**

 We have calculated the CCA value and have added this and its interpretation to the results section under Quality of Evidence and Risk of Bias.

**4. The study should provide the specific search strategy (the combination of search terms) in a supplementary file or clearly refer to where to find it.**

 The full search strategy is available in the extended data section (per the F1000 guidelines) as Supplementary Table 1. We have added a sentence in the methods section to make finding the full search strategy clearer. 

**5. The methodological quality, risk of bias, and robustness of each evidence are not reported in the Results text. It is clearer to label them alongside the evidence. It is the same for table 1 and table 5. Table 6 can be put into the supplementary file. The overall quality, risk of bias, and robustness of each evidence should be made clear in the corresponding text and tables.**

 Because quality and risk‑of‑bias ratings apply to the systematic reviews as a whole (not to each specific outcome), linking them directly to individual findings could be misleading. For this reason, we present these assessments separately and discuss their implications in the narrative synthesis.

**6. What about the robustness of each quantitative evidence? The robustness comprehensively reflects the magnitude of the p-value, the sample size, the heterogeneity, and the risk of bias. There is a simple rule for assessing robustness suggested before. Please refer to the literature below.**

1. **Aromataris E, Fernandez R, Godfrey CM, Holly C, Khalil H, Tungpunkom P. Summarizing systematic reviews: methodological development, conduct and reporting of an umbrella review approach. Int J Evid Based Healthc 2015;13:132–140.**
2. **Aromataris E, Fernandez RS, Godfrey CHolly C, Khalil H, Tungpunkom P. Methodology for JBI umbrella reviews 2014;1.**
3. **Cleophas TJ, Zwinderman AH. Meta-meta-analysis. In: Modern meta-analysis: review and update of methodologies. Cham: Springer International Publishing; 2017, p135–143.**
4. **Fusar-Poli P, Radua J. Ten simple rules for conducting umbrella reviews. Evid Based Ment Health 2018;21:95–100.**

We assessed the methodological quality and risk of bias of all included systematic reviews using both AMSTAR-2 and ROBIS. These validated tools evaluate key domains relevant to the robustness of evidence, including risk of bias, heterogeneity, and overall methodological rigour.

 The references above primarily recommend critical appraisal of the included systematic reviews and transparent reporting of heterogeneity, potential biases, and study characteristics. None of these sources explicitly mandates an additional composite "robustness" index that integrates p-value magnitude, sample size, heterogeneity, and risk of bias for each quantitative result beyond standard quality appraisal tools.

**7. Even though the included systematic reviews are different between the current umbrella review and the previous umbrella review, the included original studies (evidence) of the current umbrella review should cover most of the original studies in the previous umbrella review. Because the included systematic review did not set the criteria to include only original studies between December 2020 and June 2024, rather, they should have included earlier original studies. How do you interpret this? The readers should be aware of it when interpreting results.**

 We have added a discussion of this to the limitations section of the discussion.

**8. The current review included a wide range of diseases and various study designs. The effective exposure time of green space may vary among diseases. For example, the time scale of the association between green space and CVD is usually years, whereas the time scale of the association between green space and mental health can be days. This should be reported in tables and results. And it could be an interesting discussion point.**

 We agree that exposure timeframes may differ across diseases. However, many of the systematic reviews included in our umbrella review do not report exposure duration and deriving or assigning exposure timeframes would require re‑interpreting primary studies, which is beyond the scope of an umbrella review. For these reasons, we have not added exposure‑time information to the tables or results.

**9. What is the limitation of the current study?**

 We have added a paragraph to more explicitly discuss the limitations of this umbrella review.

**10. The authors should give more implications for policy and future studies regarding health domains, respectively.**

 While we agree that policy and research implications are important, it is difficult to provide meaningful domain‑specific implications without content analysis of existing policy worldwide.

**Minor**

**1. In the first paragraph of the Introduction, you said "However, limitations in the methodology of these umbrella reviews have resulted in some gaps in the literature." But not all of the listed reviews are umbrella review. You can consider to rephrase it to "these umbrella or systematic reviews".**

 We have adjusted the wording in this section for clarity.

**2. In the second paragraph of the Introduction, you first said "these reviews often present their findings narratively", then you gave an example review that "focused solely on quantitative results". It is not clear what you suggest from here? Should study include both quantitative and qualitative analyses? Or maybe you want to demonstrate that previous reviews did not include both quantitative and qualitative results. But you should give more example reviews.**

 This paragraph has been revised for clarity.

**3. Some texts are not well cited. For example, in the Discussion section, "Systematic reviews examining respiratory outcomes such as asthma and AR presented mixed findings – some reporting protective effects, others showing null or conflicting associations". Please examine thoroughly to make sure the texts have citations where it is necessary.**

 The statement in question summarises findings that are already fully referenced in the Results section. Because the Discussion section synthesises previously cited material rather than introducing new evidence, we have not repeated citations here. However, we have re‑checked the manuscript to ensure that all statements requiring citation are appropriately referenced.

# Reviewer report: Reviewer response for version 1

**DOI:** 10.5256/f1000research.183900.r435773

**Published:** 2025-12-24

**Authors:** David Rojas-Rueda

**Affiliations:** 1 Colorado State University, Fort Collins, USA

**Feedback for Improvement:**

 1. **Transparency of Study Design in Meta-Analyses:** The results derived from meta-analyses pooling data primarily from cross-sectional or ecological studies are highly problematic for inferring causality or defining risk, as they are "only useful to define a hypothesis, not to identify that relationship". Even though the paper provides a narrative discussion of the overall study design distribution (Table 2), Table 5 should be enhanced to specify the underlying study designs for each quantitative finding.

     ◦ For example, Bianconi et al. (2023) summarized cardiovascular outcomes pulling from 15 ecological, 13 cohort, 7 cross-sectional, and 1 case-control study. If their quantitative estimates in Table 5 are derived from this mixed pool, a risk assessor cannot easily distinguish the quality of the pooled result.

     ◦ **Recommendation:** Clarify in the text or in an expanded version of Table 5 the *type and proportion of original studies* (e.g., Cohort/RCT vs. Cross-sectional/Ecological) contributing to each meta-analysis result. This prevents the perpetuation of bias where the meta-analysis result might be interpreted as a strong causal effect when the underlying evidence base is correlational.

 B. Enhancing Dose-Response Information (Table 5)

 Table 5, listing quantitative data from meta-analyses, is the most crucial section for PRA. However, it currently lacks the standardization needed for clear risk modeling.

**Dose-Response Functions and Exposure Units:**

 1. **Dose Definition and Units:** Many entries in Table 5 rely on the **Normalised Difference Vegetation Index (NDVI)**. The protective effect should be explicitly linked to a defined unit of change in exposure (the dose).

     ◦ The table labels the Exposure Measure generally (e.g., "NDVI 500m buffer") but rarely defines the exposure increment associated with the effect estimate (e.g., OR/HR per 0.1 unit increase in NDVI).

     ◦ One example where relative dose information is present is Ahmer et al. (2024), where the **standardized regression coefficient (beta)** is listed for Birth weight (BW) relative to NDVI 250m and 500m buffers. Similarly, Zhao et al. (2022) provides standardized regression coefficients for systolic and diastolic BP in relation to "Overall greenspace" exposure.

     ◦ **Recommendation:** Systematically list the implied "dose" unit for all non-binary exposures (i.e., NDVI, Area of greenspace, etc.) in Table 5. If the original meta-analysis reported relative risk for quartile comparison, this should be noted (e.g., "Highest Quartile NDVI vs. Lowest Quartile"). If the study provided results per standard deviation or a standard incremental increase (like the 0.1-unit increment in NDVI mentioned in the narrative for asthma incidence), this information must be made explicit within the table.

 2. Missing Contextual Information (Population and Geography)

 For effective public health targeting, understanding which populations and regions derive the most benefit is essential.

 A. Population Specificity (Age Groups and Health Status)

 While the review states that it aims to encompass *all age groups* and mentions studies focused on children and adolescents and older adults, this information is not consistently integrated into the core summary tables.

 1. **Age and Health Status Context in Tables:** The utility of a finding for a risk assessor depends heavily on the population studied. For instance, the protective effect of greenspace exposure on mortality might be different in a cohort of healthy adults versus older adults with pre-existing cardiovascular conditions.

     ◦ Yuan et al. (2021) focused on mortality and cardiovascular outcomes **in older adults**.

     ◦ Ye et al. (2022) focused on health outcomes **in childhood and adolescence**.

     ◦ **Recommendation:** Systematically add a column to Table 1 (or Table 5 for quantitative data) explicitly identifying the **primary population group** (e.g., Children/Adolescents, Adults, Older Adults, Mixed) studied in the included systematic reviews. This improves contextual relevance for targeted policy recommendations.

 B. Geographical Context and Disparities

 The paper notes crucial findings regarding socioeconomic status (SES) and urbanicity, such as stronger protective associations in urban areas compared to less urban areas, and larger benefits for individuals with lower SES. This is highly important for addressing health equity (a key component of modern environmental health).

 1. **Geographical Reporting:** The review includes Rojas-Rueda et al. (2021) which specifically addresses evidence in **Latin America**. Similarly, Rahimi-Ardabili et al. (2021) covers green space and health **in Mainland China**. However, the primary geographical focus of other contributing reviews is not consistently highlighted in the data summary tables.

     ◦ **Recommendation:** Include a column in Table 1 that explicitly identifies the **geographic region** or primary focus population (e.g., China, Latin America, Global/Mixed HIC) for each included systematic review. This information is vital, allowing policymakers to evaluate generalizability.

 3. Missing Key Literature

 Rojas-Rueda D, Nieuwenhuijsen MJ, Gascon M, Perez-Leon D, Mudu P. **Green spaces and mortality: a systematic review and meta-analysis of cohort studies.** (REFER TO 1)

**Finding:** This review, published in 2019, appears to be missing from the current umbrella review, which restricted its search to articles published between **December 2020 and June 2024**.

**Critique on Exclusion:** While adherence to the established search protocol (December 2020 onward) is understandable for an *update* to a previous review, the excluded Rojas-Rueda (2019) paper is a **systematic review and meta-analysis of *cohort studies*** specifically on **mortality**, an outcome discussed in detail here. Because cohort studies provide stronger evidence for causality than cross-sectional studies (which dominate the current base), and because the authors are looking to update knowledge based on previous work (Bryer et al., 2024), it is essential to contextualize the current findings against high-quality pre-2020 meta-analyses that remain highly relevant.

**Recommendation:** The authors should clarify if the 2019 Rojas-Rueda mortality review was included in their *prior* umbrella review (Bryer et al., 2024) and, if so, explicitly reference it in the narrative (e.g., when discussing all-cause mortality findings based on cohort data) to maintain a complete historical context of the highest-quality evidence available in this domain. If this 2019 meta-analysis (focusing solely on cohort data) contradicted or strongly reinforced the results summarized from Yuan et al. (2021) and Bianconi et al. (2023), noting this distinction would significantly improve the paper's utility.

 Summary of Key Recommendations for Improving Utility

 To maximize the utility of this excellent work for public health policy, I strongly recommend the following improvements:

 1. **Enhance Table 5 (Quantitative Data):** Systematically include columns detailing:

     ◦ The **unit or increment of exposure** corresponding to the effect estimate (Dose-Response function).

     ◦ The **primary study designs** (e.g., % Cohort/RCT vs. % Cross-sectional/Ecological) underlying the meta-analysis result.

 2. **Enhance Table 1 (Review Characteristics):** Systematically include columns detailing:

     ◦ The **primary demographic group** studied (e.g., Children/Adolescents, Older Adults, Mixed Population).

     ◦ The **geographic region** or scope (e.g., China, Latin America, Global/HIC).

 3. **Narrative Contextualization:** Ensure that the discussion of high-quality pre-2020 systematic reviews (like the 2019 Rojas-Rueda mortality paper) is provided, justifying how the current "update" relates to established, high-causality evidence.

 These additions would transform the paper into a far more actionable resource for environmental epidemiologists and urban planners focused on targeted green space policies and health risk quantification.

Are the rationale for, and objectives of, the Systematic Review clearly stated?

Yes

Is the statistical analysis and its interpretation appropriate?

Partly

If this is a Living Systematic Review, is the ‘living’ method appropriate and is the search schedule clearly defined and justified? (‘Living Systematic Review’ or a variation of this term should be included in the title.)

Partly

Are sufficient details of the methods and analysis provided to allow replication by others?

Yes

Are the conclusions drawn adequately supported by the results presented in the review?

Partly

Reviewer Expertise:

Environmental epidemiology

I confirm that I have read this submission and believe that I have an appropriate level of expertise to confirm that it is of an acceptable scientific standard, however I have significant reservations, as outlined above.

## References

[1] : Green spaces and mortality: a systematic review and meta-analysis of cohort studies. *The Lancet Planetary Health* .2019;3(11) : 10.1016/S2542-5196(19)30215-3e469-e477 10.1016/S2542-5196(19)30215-3 PMC687364131777338

# Response

**Published:** 2026-03-31

**Authors:** Darsy Darssan

**Affiliations:** The University of Queensland, Australia

We are grateful for the valuable and detailed comments on our manuscript. We have thoughtfully reviewed and integrated your recommendations to the extent possible.

**Transparency of Study Design in Meta-Analyses: The results derived from meta-analyses pooling data primarily from cross-sectional or ecological studies are highly problematic for inferring causality or defining risk, as they are "only useful to define a hypothesis, not to identify that relationship". Even though the paper provides a narrative discussion of the overall study design distribution (Table 2), Table 5 should be enhanced to specify the underlying study designs for each quantitative finding.**

- **For example, Bianconi et al. (2023) summarized cardiovascular outcomes pulling from 15 ecological, 13 cohort, 7 cross-sectional, and 1 case-control study. If their quantitative estimates in Table 5 are derived from this mixed pool, a risk assessor cannot easily distinguish the quality of the pooled result.**
- **Recommendation: Clarify in the text or in an expanded version of Table 5 the type and proportion of original studies (e.g., Cohort/RCT vs. Cross-sectional/Ecological) contributing to each meta-analysis result. This prevents the perpetuation of bias where the meta-analysis result might be interpreted as a strong causal effect when the underlying evidence base is correlational.**

We have added the study designs and numbers to the third column of Table 5.

**Enhancing Dose-Response Information (Table 5)**

**Table 5, listing quantitative data from meta-analyses, is the most crucial section for PRA. However, it currently lacks the standardization needed for clear risk modeling.**

**Dose-Response Functions and Exposure Units:**

**Dose Definition and Units: Many entries in Table 5 rely on the Normalised Difference Vegetation Index (NDVI). The protective effect should be explicitly linked to a defined unit of change in exposure (the dose).**

- **The table labels the Exposure Measure generally (e.g., "NDVI 500m buffer") but rarely defines the exposure increment associated with the effect estimate (e.g., OR/HR per 0.1 unit increase in NDVI).**
- **One example where relative dose information is present is Ahmer et al. (2024), where the standardized regression coefficient (beta) is listed for Birth weight (BW) relative to NDVI 250m and 500m buffers. Similarly, Zhao et al. (2022) provides standardized regression coefficients for systolic and diastolic BP in relation to "Overall greenspace" exposure.**
- **Recommendation: Systematically list the implied "dose" unit for all non-binary exposures (i.e., NDVI, Area of greenspace, etc.) in Table 5. If the original meta-analysis reported relative risk for quartile comparison, this should be noted (e.g., "Highest Quartile NDVI vs. Lowest Quartile"). If the study provided results per standard deviation or a standard incremental increase (like the 0.1-unit increment in NDVI mentioned in the narrative for asthma incidence), this information must be made explicit within the table.**

We have added the dose or categories being compared to the fifth column of Table 5.

**Missing Contextual Information (Population and Geography)**

**For effective public health targeting, understanding which populations and regions derive the most benefit is essential.**

**Population Specificity (Age Groups and Health Status)**

**While the review states that it aims to encompass all age groups and mentions studies focused on children and adolescents and older adults, this information is not consistently integrated into the core summary tables.**

**Age and Health Status Context in Tables: The utility of a finding for a risk assessor depends heavily on the population studied. For instance, the protective effect of greenspace exposure on mortality might be different in a cohort of healthy adults versus older adults with pre-existing cardiovascular conditions.**

- **Yuan et al. (2021) focused on mortality and cardiovascular outcomes in older adults.**
- **Ye et al. (2022) focused on health outcomes in childhood and adolescence.**
- **Recommendation: Systematically add a column to Table 1 (or Table 5 for quantitative data) explicitly identifying the primary population group (e.g., Children/Adolescents, Adults, Older Adults, Mixed) studied in the included systematic reviews. This improves contextual relevance for targeted policy recommendations.**

We have added the age range of studies in each systematic review to the fifth column of Table 1.

**Geographical Context and Disparities**

**The paper notes crucial findings regarding socioeconomic status (SES) and urbanicity, such as stronger protective associations in urban areas compared to less urban areas, and larger benefits for individuals with lower SES. This is highly important for addressing health equity (a key component of modern environmental health).**

**Geographical Reporting: The review includes Rojas-Rueda et al. (2021) which specifically addresses evidence in Latin America. Similarly, Rahimi-Ardabili et al. (2021) covers green space and health in Mainland China. However, the primary geographical focus of other contributing reviews is not consistently highlighted in the data summary tables.**

- **Recommendation: Include a column in Table 1 that explicitly identifies the geographic region or primary focus population (e.g., China, Latin America, Global/Mixed HIC) for each included systematic review. This information is vital, allowing policymakers to evaluate generalizability.**

We have added the location(s) covered by each systematic review to the second column of Table 1. Due to the large number and wide variety of countries covered by systematic reviews, we chose to report study location by continent instead.

**Missing Key Literature**

**Rojas-Rueda D, Nieuwenhuijsen MJ, Gascon M, Perez-Leon D, Mudu P. Green spaces and mortality: a systematic review and meta-analysis of cohort studies. (REFER TO 1)**

**Finding: This review, published in 2019, appears to be missing from the current umbrella review, which restricted its search to articles published between December 2020 and June 2024.**

**Critique on Exclusion: While adherence to the established search protocol (December 2020 onward) is understandable for an update to a previous review, the excluded Rojas-Rueda (2019) paper is a systematic review and meta-analysis of cohort studies specifically on mortality, an outcome discussed in detail here. Because cohort studies provide stronger evidence for causality than cross-sectional studies (which dominate the current base), and because the authors are looking to update knowledge based on previous work (Bryer et al., 2024), it is essential to contextualize the current findings against high-quality pre-2020 meta-analyses that remain highly relevant.**

- **Recommendation: The authors should clarify if the 2019 Rojas-Rueda mortality review was included in their prior umbrella review (Bryer et al., 2024) and, if so, explicitly reference it in the narrative (e.g., when discussing all-cause mortality findings based on cohort data) to maintain a complete historical context of the highest-quality evidence available in this domain. If this 2019 meta-analysis (focusing solely on cohort data) contradicted or strongly reinforced the results summarized from Yuan et al. (2021) and Bianconi et al. (2023), noting this distinction would significantly improve the paper's utility. **

The scope of this umbrella review was restricted to systematic reviews published between December 2020 and June 2024. As a result, the systematic review by Rojas-Rueda et al. (2019) fell outside the predefined eligibility window and was therefore not included. The study by Rojas-Rueda et al. (2019) was, however, included in our previous umbrella review, which considered systematic reviews published between January 2010 and December 2020. Because this is not a living review and the time window was established a priori, we did not re-include studies published before December 2020 in this umbrella review.

**Summary of Key Recommendations for Improving Utility**

**To maximize the utility of this excellent work for public health policy, I strongly recommend the following improvements:**

1. **Enhance Table 5 (Quantitative Data): Systematically include columns detailing:**
  1. **The unit or increment of exposure corresponding to the effect estimate (Dose-Response function).**
  2. **The primary study designs (e.g., % Cohort/RCT vs. % Cross-sectional/Ecological) underlying the meta-analysis result.**
2. **Enhance Table 1 (Review Characteristics): Systematically include columns detailing:**
  1. **The primary demographic group studied (e.g., Children/Adolescents, Older Adults, Mixed Population).**
  2. **The geographic region or scope (e.g., China, Latin America, Global/HIC).**
3. **Narrative Contextualization: Ensure that the discussion of high-quality pre-2020 systematic reviews (like the 2019 Rojas-Rueda mortality paper) is provided, justifying how the current "update" relates to established, high-causality evidence.**

**These additions would transform the paper into a far more actionable resource for environmental epidemiologists and urban planners focused on targeted green space policies and health risk quantification.**

**References**

**1. Rojas-Rueda D, Nieuwenhuijsen M, Gascon M, Perez-Leon D, et al.: Green spaces and mortality: a systematic review and meta-analysis of cohort studies. The Lancet Planetary Health. 2019; 3 (11): e469-e477**

 Thank you for summarising your recommendations. We have responded to each comment and recommendation separately above.
